# Magnetic assessment and modelling of the Aramis undulator beamline

**DOI:** 10.1107/S1600577518002205

**Published:** 2018-04-03

**Authors:** M. Calvi, C. Camenzuli, R. Ganter, N. Sammut, Th. Schmidt

**Affiliations:** a Paul Scherrer Institute, CH-5232 Villigen PSI, Switzerland; b Department of Microelectronics and Nanoelectronics, University of Malta, Msida, Malta

**Keywords:** hard X-ray line, in-vacuum undulator, magnetic measurements

## Abstract

Within the SwissFEL project at the Paul Scherrer Institute (PSI), the hard X-ray line (Aramis) has been equipped with short-period in-vacuum undulators, known as the U15 series. The undulator design has been developed within the institute itself, while the prototyping and the series production have been implemented through a close collaboration with a Swiss industrial partner, Max Daetwyler AG, and several subcontractors. The magnetic measurement system has been built at PSI, together with all the data analysis tools. The Hall probe has been designed for PSI by the Swiss company SENIS. In this paper the general concepts of both the mechanical and the magnetic properties of the U15 series of undulators are presented. A description of the magnetic measurement equipment is given and the results of the magnetic measurement campaign are reported. Lastly, the data reduction methods and the associated models are presented and their actual implementation in the control system is detailed.

## Abstract 2

The design, the magnetic optimisation and the characterisation of the Aramis undulators are presented. The SUBLIME model integrates all the information gained during the series production and the test campaign to operate the Aramis undulator beamline, including phase matching and orbit correction.

## 1. Introduction   

As part of the general strategy of the Paul Scherrer Institute (PSI) regarding the development of light sources for research, a compact free-electron laser (FEL) called SwissFEL has been designed and constructed (Milne *et al.*, 2017 ▸).

**Figure 1:**
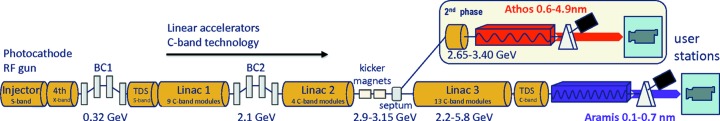
SwissFEL accelerator layout, consisting of an S-band injector, a C-band linear accelerator and two undulator beamlines. The magnetic measurements, optimization and modelling of the Aramis beamline are described in the following sections.

**Figure 2:**
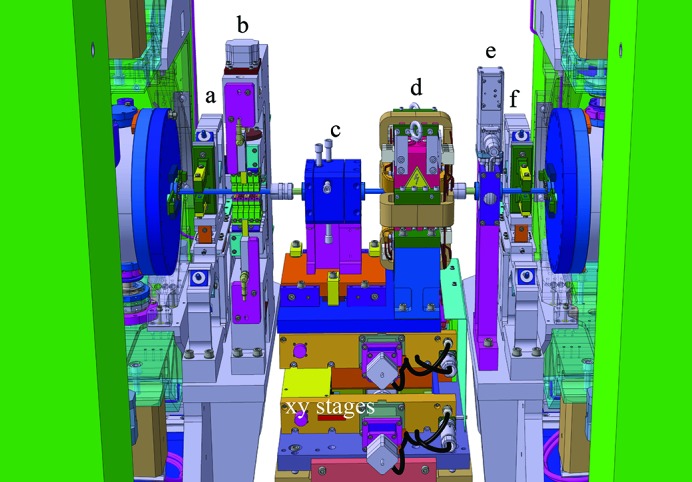
Three-dimensional drawing of the intra-undulator section. Right after the upstream undulator are the alignment quadrupole (*a*), Qal, the phase shifter (*b*), the RF beam-position monitor (BPM) (*c*), the main quadrupole (*d*) with its correction coils, the vacuum valve (*e*) and the alignment quadrupole of the downstream undulator (*f*). The Qal and the phase shifter are attached to the upstream undulator; the valve and the second Qal to the downstream one; while the BPM and the quadrupole are on a separated support, where they can be moved in the *xy*-plane.

SwissFEL consists of a low-emittance injector (Schietinger *et al.*, 2016 ▸), a linac based on C-band accelerating technology and two beamlines: a soft X-ray beamline, Athos, which is under construction, covering the photon wavelength range between 0.6 and 4.9 nm, and a hard X-ray beamline, Aramis, which is under commissioning, covering the wavelength range between 1 and 7 Å (see Fig. 1 ▸). Short-period in-vacuum undulators have been designed and installed within Aramis to achieve short emission wavelengths down to the interatomic scale with relatively low electron energies (see Table 1 ▸). Their magnetic structure has been designed only for on-axis operation, enough for a linac-driven FEL, thus reducing the magnetic forces while enhancing the field on the magnetic axis (see §2 for more details). To compromise between the total length of the beamline and the logistics of a single module, a length of 4.0 m has been selected. A distance of about 0.75 m between each pair of modules has been allocated for the installation of focusing elements, phase shifters, alignment devices and beam diagnostics (see Fig. 2 ▸ for more details).

The modelling of the undulator beamline will be addressed in detail following a description of the U15 design and a summary of the magnetic measurement results. The phase shifters will then be discussed since they are essential to be able to operate the different modules together as a single long undulator as well as the active feed-forward orbit correction scheme based on the results of the magnetic measurements. This complex multi-system model is referred to as SUBLIME (aramiS Undulator BeamLIne ModEl) and summarizes all the information that is required to operate the Aramis undulator from the control room.

## 2. U15 design   

The U15 series is made up of in-vacuum undulators with a period length of 15.0 mm. They are each equipped with a *gap drive system* which varies the *K*-value by changing the distance between the upper and the lower magnetic arrays. The minimum gap is designed to be 3.0 mm, corresponding to a *K*-value of about 1.8, and the maximum gap is about 20.0 mm, which is enough to reduce the *K*-value below 0.05.

**Figure 3:**
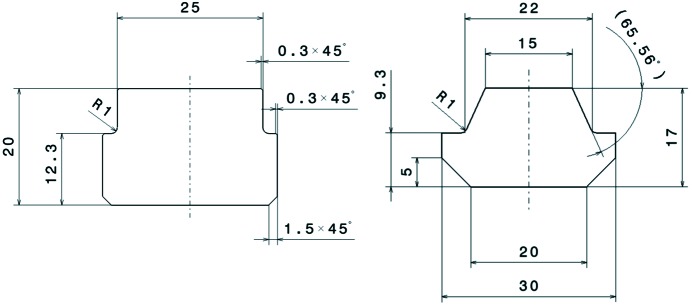
Two-dimensional drawings of the U15 magnets (left) and the U15 poles (right). The thickness of the magnets and of the poles is 5.0 and 2.4 mm, respectively. Dimensions are given in units of millimetres.

The magnetic structure consists of NdFeB (*B*_r_ = 1.25 T and *H*_cj_ > 2300 kA m^−1^, achieved with the Dy diffusion process) permanent magnets and FeCo (*B*_sat_ = 2.35 T) poles in order to achieve the highest field on the beam axis (see Fig. 3 ▸). Since the electron bunch travels through the undulator line only once, the requirements concerning lifetime and instabilities that have to be considered for storage rings are not significant. The pole horizontal width can therefore be reduced to a tip of only 15.0 mm, as opposed to, for instance, 40.0 mm as is used in the in-vacuum undulator found in the Swiss Light Source storage ring. This choice decreases the magnetic forces from about 70 kN down to 27 kN, substantially reducing the deformation of the mechanical part and thus improving the overall accuracy of the field profile.

**Figure 4:**
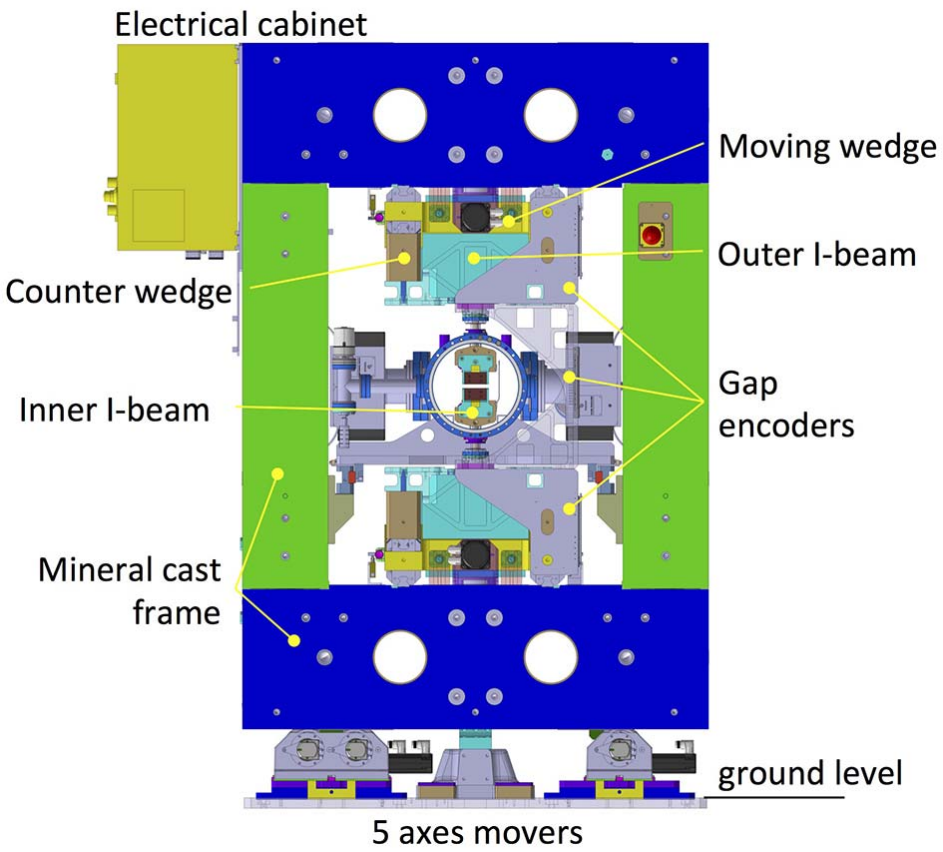
Sketch of the U15 cross section highlighting its main components. The closed frame, which is fabricated from mineral cast, is made of two parts: the bottom and the top sides (in blue) and the left and the right sides (in green). The latter supports the vacuum chamber. The moving wedge (in yellow), the counter wedge (in light brown) and the outer I-beam (in light blue) together form the *gap drive system*, the position of which is precisely controlled with three linear encoders. Inside the vacuum chamber, the inner I-beam supports the magnetic structure. This is pre-assembled into a series of aluminium extruded elements, referred to as *block-keepers*. The electrical cabinet (top left, in yellow) houses the control system and the power supplies of all servo motors (in black). The U15 undulator rests on a cam shaft mover system (on the bottom) which can displace the undulator in the vertical and horizontal direction, as well as orient it in the three Euler angles (pitch, yaw and roll). It is fixed on plates that are integrated below ground level.

To further improve the stiffness of the device, a closed-frame solution has been selected in place of the more popular C frame (see Fig. 4 ▸). This was possible thanks to the SAFALI (Tanaka *et al.*, 2007 ▸) magnetic measuring bench concept (see §3), originally developed for cryogenic in-vacuum undulators (Hara *et al.*, 2004 ▸; Calvi *et al.*, 2013 ▸), which is no longer based on the straightness of an external reference bench (traditionally a large, heavy and stable granite bench). Cast mineral is used for the frame material, which is quite original for undulator applications where cast iron is regularly used. This material has superior damping properties which makes it popular for high-precision milling and grinding machines, and for its application in the undulator it holds the remarkable properties of being almost non-magnetic (μ_r_ ≃ 1). However the decision to opt for this configuration was mainly driven by the cost optimization study. This technique has proven to be cost effective on a small series production like the 13 units of the Aramis line. Thanks to the symmetry of the structure, two moulds were enough for the full production. The overall process is performed at room temperature since there is no need to heat up the material, thus saving energy and cost. Additionally, it is possible to modulate the weight and the shape of these casts with simple techniques at low cost.

**Figure 5:**
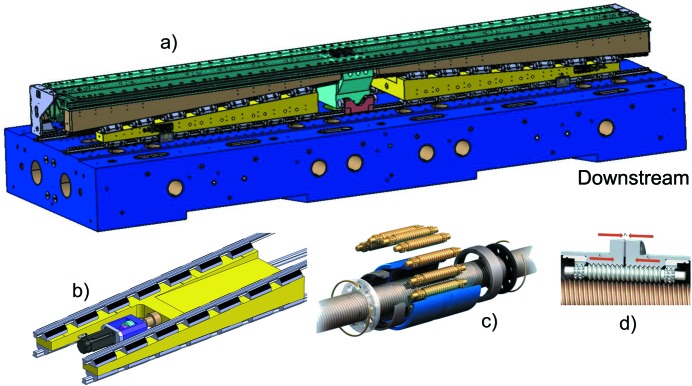
(*a*) Base frame (which is identical to the upper frame) and the two moving wedges (in yellow) that are connected with a precise and stiff guiding system, connected to both the bottom mineral cast frame and to the outer I-beam, where the counter wedges are assembled. The central bearing system that prevents erroneous longitudinal displacement can be seen in the middle (light blue). (*b*) Detail of the moving wedge (in yellow). This is driven by a servo motor (in black), fixed on a nut (in purple) and attached to the frame (on its bottom side). (*c*) Satellite roller screws used to drive the spindle. (*d*) Pre-loading of a satellite roller screw used to minimize the backlash.

To cope with the more stringent requirements for *K*-value control (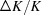 < 10^−4^), a new gap drive system has been designed to reach sub-micrometre reproducibility levels. It is based on opposing wedges angled at 3° (see Fig. 5 ▸). This allows the system to be moved without the assistance of a gear box because the wedge operates with a ratio of about 19 between the longitudinal and vertical displacement. A special spindle with a pitch of only 1 mm per turn, equipped with pre-compressed satellite roller screws, is implemented to minimize the backlash. Moreover, the wedge system has a second but equally important functionality: transferring the stiffness of the mineral cast frame to the magnetic arrays. Over a length of 4.0 m, it is not easy to manufacture a single long wedge with the required accuracy and, in the specific case of the U15 series, the height of the wedge would not be compatible with the size of the frame and with the beam height. Therefore, the solution for U15 utilizes two wedges per magnetic array, which are referred to as upstream and downstream wedges. The two fixed wedges are connected through the so-called outer I-beam. This requires the two wedges to move synchronously, otherwise the outer I-beam would bend and possibly deform permanently. Finally, the two moving wedges have been synchronized *via* the real-time bus of the control system, based on the reading of two linear encoders, installed upstream and downstream, respectively. These encoders read the distance of the outer I-beam with respect to the bottom and the top frame, respectively. Four encoders give the position of the four wedges for any given gap value. Two additional encoders have been installed for diagnostic purposes and read the distance between the top and bottom outer I-beams (see Fig. 4 ▸).

During regular operation, this option is preferred to a mechanical connection through a long shaft but it is prone to rare but fatal error in the electronics. To prevent such a possible scenario, an additional protection system has been implemented to monitor the status of the wedge (suggested by an external committee during the review meeting after the manufacture of the prototype). This works by means of a set of micro-switches that disconnect the power of the servo motors if their position differs by more than 50 µm. Up to this amount, deformation is acceptable because it is still fully reversible.

**Figure 6:**
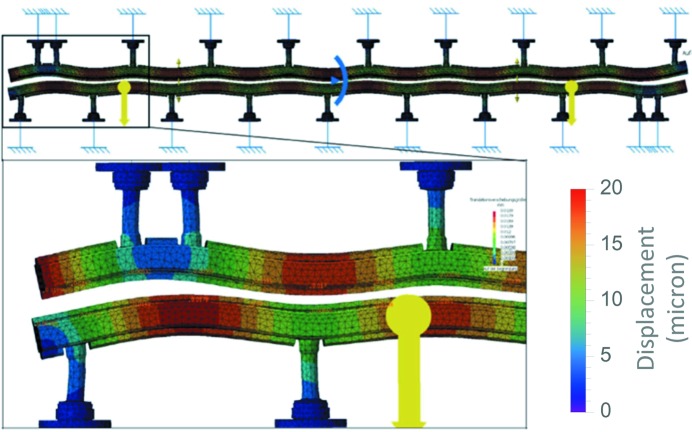
The top part of the illustration shows the full length of the in-vacuum components which are under magnetic force. The two inner I-beams are fixed to a set of columns arranged specifically to minimize the changes in the longitudinal profile of their relative distance, *i.e.* the gap profile. The lower part of the figure details the simulation where the absolute displacement is presented with a chromatic scale calibrated on the right.

The two magnetic arrays are assembled inside the vacuum chamber and are connected to the outer I-beam through a mechanical feed-through, which for simplicity is referred to as the *column* throughout this paper. The number and the position of these columns have been optimized with finite-element method (FEM) calculations to minimize the amount of columns to be implemented on each undulator module, while keeping the deformation produced by the magnetic forces within a reasonable range. The retained solution is sketched in Fig. 6 ▸.

In the former in-vacuum undulator design (Hara *et al.*, 1998 ▸; Schmidt *et al.*, 2001 ▸), two columns were always used to hold the I-beam in one cross section. Counting both upper and lower I-beams, there are four columns in one cross section. This is essential to precisely control the angle between the upper and the lower I-beams. As was discussed previously for the magnetic design, the out-of-axis magnetic field does not affect the performance of SwissFEL as is the case for a storage ring. This allows the replacement of the two-column system with a single one, yet still designed to withstand the same forces. This change reduces the time required for the optimization, not only because of the reduced number of components but also due to the intrinsic difficulty of manipulating two columns that act almost on the same mechanical point.

The upper and the lower columns have not been placed at the same position but have been longitudinally shifted by half of the distance between two adjacent columns attached to the same array. This simple strategy allows the number of columns to be reduced while ensuring that the distance between the lower and the upper array (*i.e.* the longitudinal gap profile) is only marginally modified by the magnetic forces. An ANSYS calculation, see Fig. 6 ▸, predicted changes in the gap profile of about ±1 µm when varying the magnetic forces from 0 to 2.7 t. This happens while deformations up to ten times larger are accepted along the array itself, corresponding to comparable axis deviations. This is compatible with the overall accuracy because the magnetic field on-axis is exponentially sensitive to the gap, while the field varies only like a hyperbolic cosine as it moves out of the axis. The positioning of the last two columns downstream on the upper array and downstream on the lower array has been determined through a long computer simulation section to minimize problems caused by the broken periodicity.

**Figure 7:**
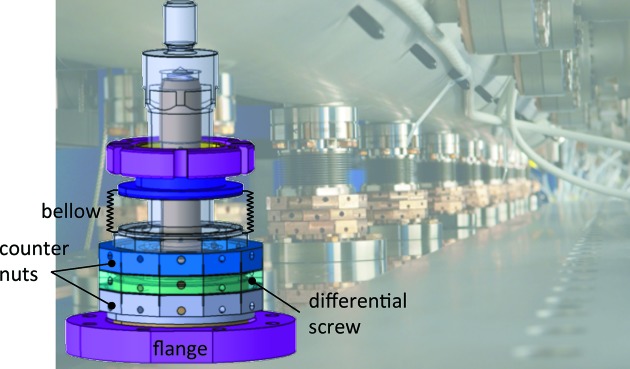
Three-dimensional drawing of the final column design. On the bottom is the flange to fix on the outer I-beam, the differential screw with the upper and lower counter-nuts, and the bellow which has been integrated in the column design to reduce the total height.

A bellow is integrated with each column to retain the vacuum while the column is moved relative to the vacuum chamber. This feature is naturally required to change the gap. Additionally, each column is equipped with a differential screw to vary the column height with micrometre precision (see Fig. 7 ▸). This is required for the coarse tuning of the magnetic field. Changing the column height locally varies the magnetic field strength, as shown in detail in §4. This functionality comes with a penalty in the stiffness of the structure. To overcome this problem, two counter-nuts have been added at the two ends of the differential screw to minimize the play in the threads as well as to prevent any relaxation in the column length after several years of operation.

**Figure 8:**
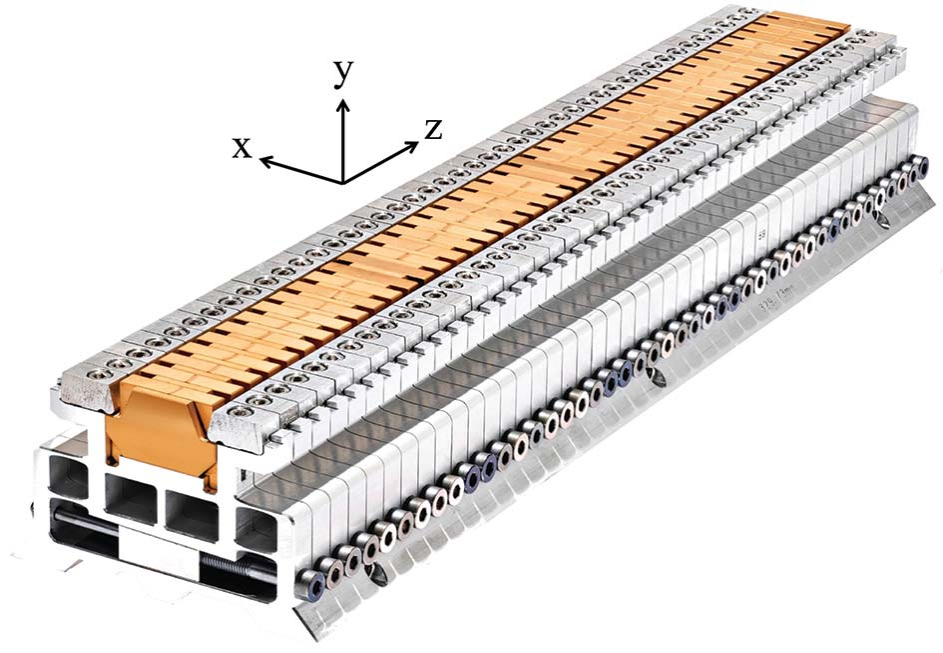
Photograph of a block keeper. The magnetic structure, which is coated with TiNi (in bronze colour), starts with a CoFe pole and ends with a NdFeB magnet. It host 22 periods (44 poles and 44 magnets). Each magnet-pole pair can be vertically displaced by ±30 µm with the help of a flexor moved by a wedge driven by a screw, as can be seen in the front cross section of the above picture. Copyright of Scanderbeg Sauer Photography.

The columns hold an aluminium profile inside the vacuum chamber, referred to as the inner I-beam, which supports the magnetic array (see Fig. 4 ▸). The magnets and the poles are assembled into an aluminium structure called the *block keeper*, where they are secured with clamps and screws. The block keeper is designed to adjust the pole height within a short range of ±30 µm. This is essential to compensate for the natural scattering in the field strength of each magnet and restrict the RMS phase error to a few degrees (see §5). Fig. 8 ▸ outlines the technical solution that is implemented for the pole height adjustment. It is based on a flexible mechanical system, in short called a *flexor*, behaving like a spring. Each flexor unit carries one pole and one magnet and it is vertically positioned with the help of a wedge (2° angle) horizontally displaced with a screw. To prevent the displacement of the poles with the changing magnetic field, the flexor is pre-loaded with enough force to always contrast the magnetic forces and avoid any motions. This is achieved with a wedge displacement that is equivalent to a pole height change of +60 µm. The magnets and the poles are covered with copper–nickel foil (50 µm Cu and 50 µm Ni) to decrease the impedance (Hara *et al.*, 1998 ▸). The roughness of the copper surface has been measured in several samples and its RMS value was found to be between 100 and 120 nm. The nickel side is used to hold the foil on the poles, due to its magnetic properties.

## 3. Magnetic measurement system   

Two new magnetic measurement benches are required for the optimization and the characterization of the undulator magnetic profile. Bench A, carrying the tuning robot unit, can be operated only without the vacuum chamber. For this reason a second bench, Bench B, was designed for the characterization of the undulator after assembling the vacuum components. The two benches are operated in two separated rooms. They are based on a three-axis Hall probe designed by the company SENIS specifically for the SwissFEL project, now available in the catalog with the reference ‘Hall sensor S’. Three Hall sensors are aligned in the direction of the beam axis (*z*-axis, which is also the direction of the probe displacement during the measurements) and oriented in the three orthogonal directions. They are spaced at 2 mm along the *z*-axis and their active area is all at the same height (*y*) and at the same horizontal position (*x*), such that the three components of the magnetic field can be measured along the same line during one longitudinal scan. The vertical (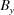) and the horizontal (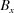) components of the magnetic field give essential information about the electron beam orbit and phase. The longitudinal field component (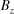) is very practical when it comes to aligning the undulator and to precisely measuring the undulator axis profile (along the beam axis), which might not be a simple straight line. This method has been implemented for the first time during this measurement campaign and it will be clarified further in §4.

The SENIS probe comes with electronics that implement the spinning current technique (Popovic *et al.*, 2007 ▸; Popovic, 2014 ▸) to minimize the Hall planar effect (Popovic, 2003 ▸). The probe provides calibrated and temperature-compensated analog signals that are proportional to the three components of the magnetic field. Digitization and synchronization with the encoder measuring the position of the probe along the undulator is made with the industrial PC-based Beckhoff PLC. This system implements the real-time EtherCAT protocol to read the information collected among the different cards, which allows easy synchronization between the motion control and the data acquisition. After measuring the noise and linearity, the Beckhoff ADCs (16-bit and 10 kHz) have been implemented and carefully synchronized to the encoder. There is an option available for some card families, known as distribution clocks, which allow the minimization of jitter among a group of cards. This is achieved within a bus clock, specifically 0.5 ms. With this technique it was possible to synchronize the three ADCs and the longitudinal Heidenhein encoder better than 0.01 ms without any additional trigger signal.

**Figure 9:**
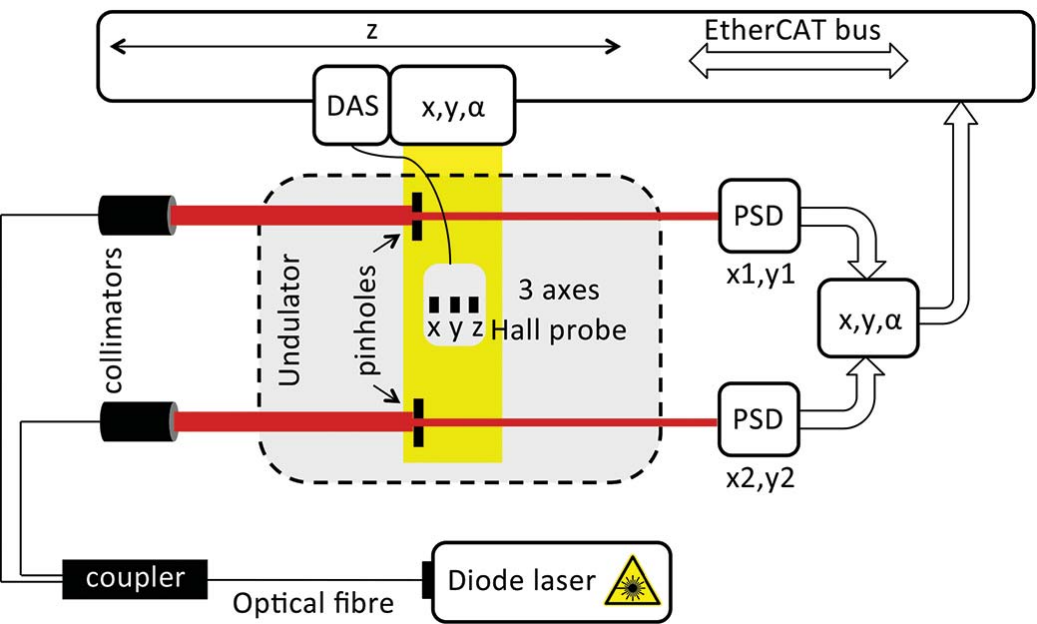
Schematic view of magnetic measurement Bench A. A diode laser generates the red beam that, after splitting, stabilizes the transverse position of the Hall probe. While a linear motor moves the probe along the undulator, the laser signals out of the two pinholes attached to the probe are used to correct its position within ±20 µm.

Both benches displace the probe at 10 mm s^−1^ along a straight line by means of an active transversal stabilization system based on SAFALI (Tanaka *et al.*, 2007 ▸) (see Fig. 9 ▸). The working principle is as follows: a laser beam shines on a pinhole which is rigidly connected to the Hall probe. The fraction of light passing through the pinhole travels downstream, where its transversal position is measured with a position-sensitive diode (PSD; made with four quadrant diodes). The SwissFEL measuring bench is equipped with two laser beams and a pinhole on each of the two sides of the probe. This allows for the accurate measurement of both the position and the angle of the probe and actively corrects its deviation from a straight line. To precisely measure the Earth’s magnetic field and the first field integrals, a moving wire system is used together with the Hall probe. The moving wire system consists of a CuBe wire of 120 µm diameter and is moved at a constant speed of 5 mm s^−1^ with a set of servo motors. The stray field of the servo motor is shielded with μ-metal cups, otherwise visible as an AC signal across the moving wire. The details of the analysis procedure are provided in §4. An alternative procedure for the evaluation of the Earth field directly with the Hall probe is to implement the zero gauss chamber, where the electronic offset can be precisely measured. This approach was used to validate the moving wire but not used on the series measurement tests.

The reproducibility of the two benches has been estimated and presented in Table 2 ▸. Two definitions have been used, called short-term and long-term reproducibility, respectively. The short-term is defined as the difference between two consecutive measurements of the same undulator without any setting changes in between readings. The long-term is defined among 100 consecutive measurements (about 24 h). Two parameters have been used, the *K*-value and the RMS phase error, Δϕ_err_. For the short-term, the reproducibility of the *K*-value is almost identical on both benches and equal to 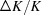 ≃ 0.3 × 10^−4^ (measured at *K* = 1.52). On the contrary, the RMS phase error is more reproducible on Bench B (∼0.2°) than on Bench A (∼0.7°). For the long-term reproducibility, Bench B is superior: 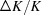 ≃ 0.6 × 10^−4^ against 2.1 × 10^−4^ of Bench A. The long-term reproducibility of the RMS phase error on the two benches is identical to the short-term one. The higher long-term reproducibility of Bench B is due to the better temperature stabilization of the room: due to its smaller dimensions and to the less frequent human activities. The higher reproducibility of Bench B (both the short- and the long-term) in measuring the phase error is due to the lower uncertainty in the positioning of the probe in the *xy* plane (∼60 µm on Bench A and ∼20 µm on Bench B). This is a direct consequence of the lower jitter in the laser, due to the presence of the vacuum chamber which limits the air motion.

The accuracy of the measurement of the field is limited by the calibration of the SENIS probe. It is sold with a 0.25% accuracy over the full range of magnetic field (±2 T) and temperature (25 ± 20°C).

### 3.1. Measuring Bench A: undulator optimization   

Bench A consists of a linear motor displacing both the measuring head and the tuning robot along the length of the undulator (*z*-axis). The measuring head includes the SENIS Hall probe, its electronics and a set of ADCs to digitize the signal right at the probe, thus minimizing cable length and reducing the noise. It is motorized to follow the two laser signals and can be displaced vertically, laterally and in the roll angle with respect to the direction of motion.

The tuning robot consists of a motorized screwdriver that is used to adjust the pole height position. It is designed to reach both the lower and the upper arrays with the assistance of a vertical stage. While it is positioned around a given pole, a set of pneumatic cylinders moves the tool closer to the target screw. When a calibrated limit switch turns on, the system acknowledges the fact that the tool is engaged into the screw and the calculated angle of rotation required to correct the pole height is applied. If the limit switch does not turn on, a searching algorithm is activated. The driver mechanism operates along different axes independently and sequentially, namely by first changing the phase of the tool, then the height and finally the longitudinal position around the target value. After an initial run, the coordinates of all screws would be known and are saved in the memory for faster additional optimization runs.

### 3.2. Measuring Bench B: undulator characterization   

Bench B is designed to measure the magnetic field when all vacuum components are assembled but the bench is still operated in air. The main physical constraint is the vacuum chamber because it reduces the available volume for the measuring head. For this reason, the linear motor used on Bench A is replaced by a smaller piezo motor that minimizes the dimensions of the rail support. The motors of Bench A, which are used to displace the probe in the transversal plane, were substituted with a new set of motors moving the entire rail from outside the chamber, holding it along its length at six points. To reduce the complexity of the installation, the roll stage correction was not integrated and the angle was measured and mechanically adjusted to limit its deviation within a milliradian along the measurement length. Due to space restrictions, the ADCs cannot fit inside the vacuum chamber either. To overcome this issue, a long cable was integrated on the probe with the purpose of transferring the analog signal out of the chamber to be recorded. Despite all the previously mentioned limitations, the reproducibility of Bench B is superior to the one measured on Bench A. This confirms that the main source of uncertainty of the SAFALI system is the pointing stability of the laser. The presence of the vacuum chamber limits the air motion and consequently improves the laser pointing stability, thus reducing noise and improving the reproducibility of the system. The increased number of motors and encoders require more care to avoid temperature drift and temperature gradients within the magnetic structure. To remove the heat produced, all motors are actively cooled with force flow water while its temperature is controlled within a feedback loop, where the temperature of the near undulator component is stabilized. Operating the bench without the active cooling system introduces large systematic errors due to the temperature drift of the undulator magnetic structure and due to the bending of the laser beam, up to a few tenths of a millimetre over the full length of the bench, attributable to vertical temperature gradients (Schricker, 2001 ▸).

## 4. Data analysis   

Both measuring benches are designed to provide the same information: the three components of the magnetic field as a function of the probe’s position along the undulator. For the analysis’ sake, the data provided by both benches can be considered equivalent. The analysis procedure can be conveniently divided into four main steps: the raw data treatment, the alignment strategy, the data reduction and the optimization algorithm. The following sections will give a detailed overview of each aforementioned step.

### 4.1. Raw data treatment   

The three components of the magnetic field (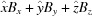) and the longitudinal encoder value (*z*) are recorded synchronously and at a frequency of 2.0 kHz. The first data conditioning consists of expressing the field as a function of the longitudinal position then filtering (in space), interpolating and uniformly re-sampling it at uniform spacing (0.5 mm).

The calibration (magnetic field *versus* voltage) is then applied to the signal. Specifically, the SENIS electronics provide a voltage signal that is already linearly correlated to the magnetic field within a calibration accuracy of 0.25% (5 V corresponds to 1 T). The electronic offsets (measured out of the undulator gap) are set to the actual Earth magnetic field value. This is estimated with the moving wire, which gives an accurate (∼1 µT) average value of the Earth field along the measuring bench, both in the 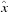 and 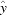 directions. The *z* component, as clarified later in §4.2, is only used for alignment purposes and does not require the Earth field correction.

By defining 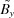 as the raw component of the vertical magnetic field, it is possible to evaluate its final corrected value, 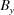, with equation (1) below,
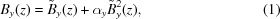
where the coefficient 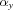 is calculated using the field integral, 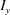 (measured with the moving wire), as shown in equation (2),
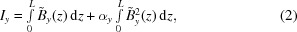
where *z* = 0 is the beginning of the Hall probe measurement and *z* = *L* (about 5 m) is the end, as is the case throughout this article. The same applies also for the *x* component of the magnetic field, 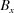. This is done to correct the calibration errors which produce a small but not negligible imbalance between the positive and negative fields, resulting in a field integral error, usually limited within 200 G cm.

### 4.2. Alignment   

The alignment of the undulator in the *x* direction and in the yaw angle is performed using only mechanical references as long as they are not critical. On the contrary, it is important to precisely orient the bench in the vertical plane (*y* and pitch) because the field varies more rapidly while moving out of the magnetic axis in the *y* direction with respect to the *x* direction. To measure a relative variation of the field of the order of 10^−4^ a movement of some millimetres is needed in the *x* direction, while it is enough to move by 40 µm in the *y* direction.

Regularly in a planar undulator, the alignment is performed by considering the vertical component of the magnetic field, 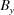, or, equivalently, the *K*-value, which is the average magnetic value along the undulator length. Repeating this measurement for different heights produces a parabola, where its local minimum is the undulator axis. More information can be extracted using the local definition of *K* (see §4.4.1) and repeating the previous analysis for each pole. This is a robust and consistent approach but is time consuming. About ten measurements are required to precisely identify the undulator axis. An alternative approach based on a new analysis of the longitudinal fields, 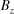, is used to measure the axis. This approach has the double advantage of reducing the alignment time while also giving an estimation of the longitudinal axis profile for each measurement. This last feature increases the reliability of the measurement campaign because it verifies whether or not each measurement is performed on-axis. Since one measurement campaign can last several working days, with the measurement equipment usually automatized to run during nights and weekends, it is essential to verify the alignment in case of arguable results.

The main advantage of this approach comes from the different definition of the axis. For the traditional method the axis is defined as 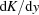 = 0, while in this approach the axis is defined as the instance where 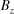 = 0. Despite its conceptual simplicity, its technical implementation is not straightforward. The first issue is the Hall planar effect. Since 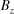 is in the background of a strong 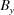, this can severely compromise the results of the analysis. The second issue is related to the geometry of the probe and the relative angle deviation between the 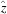 and the 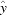 component.

**Figure 10:**
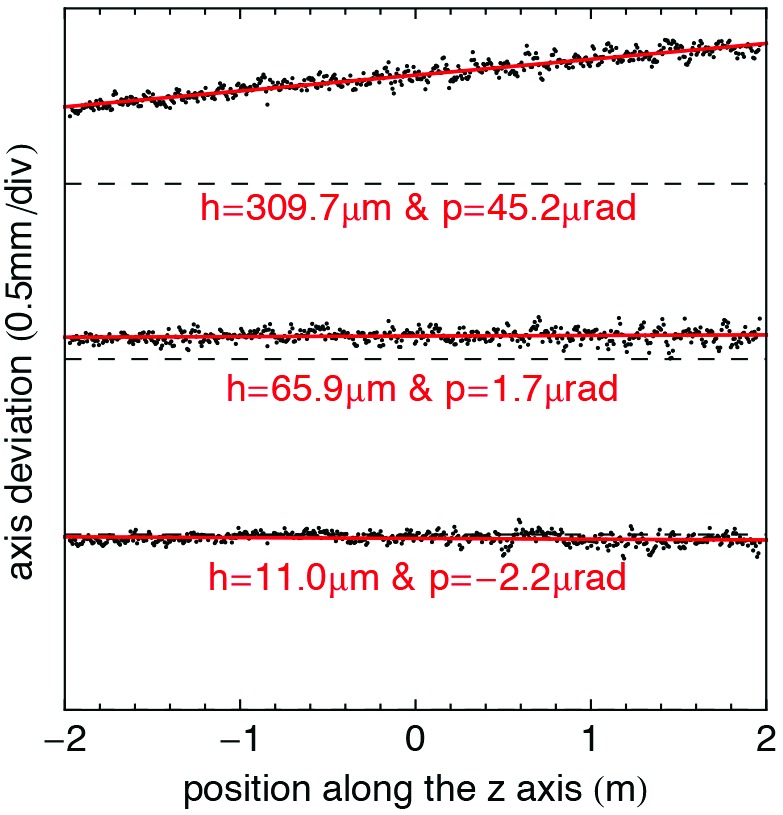
Example of magnetic axis measurements during the alignment of a U15 module on the bench. The actual axis profiles (in black) are defined with the zeros of the longitudinal field component, and the linear fits (in red) estimate the vertical offset (*h*) and the pitch angle (*p*) with respect to the measurement axis (dashed lines).

The first issue that is related to the Hall planar effect is minimized by the tri-axial SENIS probe where a four-stage spinning current method was carefully implemented to minimize the Hall planar effect and keep the noise level to a minimum. This approach also has the advantage of minimizing the offset drift thanks to polarity inversion, thus allowing longer measuring times. The second issue that concerns the geometry of the probe can be overcome with a post-processing analysis. Assuming no Hall planar effect, the angle error results in a projection of the main field, 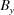, onto the 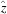 axis. For the ideal case of perfect orthogonality amongst the three components, equation (3) holds as follows,
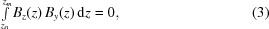
where 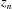 and 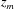 are arbitrary zeros of the main field component, 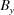, in the periodic part of the field. It is then possible to estimate the actual angle error and to compensate the signal with the following equation (4),
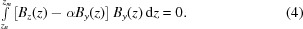
For convenience, defining the compensated field with the auxiliary variable 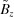 = 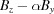 and defining the local axis as the variable 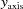 leads to equation (5),
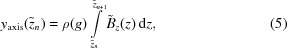
where 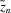 = 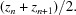 The parameter ρ is a function of the gap, *g*, and is calculated *via* a computer simulation using *RADIA* and is precisely calibrated experimentally, *i.e.* after applying a known vertical displacement of the undulator it is possible to correlate it with the results of equation (5). In Fig. 10 ▸, an example of this analysis is presented. With this information, the undulator can be aligned by means of the five-axial cam mover system in both height and pitch. Since this analysis also gives access to the axis profile, it is also possible to optimize its shape. This optimization is presented in §4.4.

This method has been benchmarked against the traditional 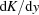 = 0 method and a discrepancy of less than 10 µm has been observed. This was tested for each probe implemented in the test campaign and the small but measurable differences among probes have been interpreted as the natural tolerances of the relative position of the sensitive area of the *z* and *y* probe. Finally, it is important to remark that the positioning of three discrete hall sensors within those slight tolerances is a remarkable achievement of the SENIS company.

### 4.3. Data reduction   

The main parameter for an undulator is the *K*-value, which gives a measurement of the electron deflection angle when divided by the relativistic Lorentz factor, γ. It is defined by the following equation, 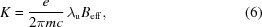
where the constant term is composed of the charge (*e*) and the mass (*m*) of the electron, and the speed of light (*c*), while the parameters 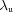 and 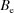 are the undulator period and the undulator magnetic field amplitude, respectively. This latter has to be evaluated for a periodic signal (see Appendix *A* [App appa]) to be within the accuracy specified for a FEL. At small gap (<4 mm), the deviation from a sinusoidal profile is visible without any Fourier analysis. The solution is as follows in equation (7),
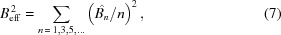
where 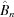 is the *n*th Fourier component and *n* can only be an odd number. For the majority of short-period in-vacuum undulators, including the U15 series, the profile is quasi-sinusoidal and the first three components of the series are enough to satisfy the fundamental undulator equation expressed in equation (8) below better than 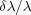 < 10^−4^,
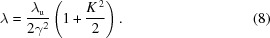
The average deflection strength is evaluated with the measurement of *K*. However, the deviation from perfect periodicity can cause severe limitations in the interference pattern of the radiation. The traditional parameter that is used to characterize the degree of spectral quality is the RMS phase error. The phase, 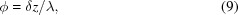
in an undulator is defined as the distance 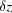 between an electron and a photon travelling along the axis of the undulator, normalized to λ, the wavelength of the first harmonic. This definition is used throughout this article and implies that each period ϕ increases by 1. Only during the evaluation of the RMS phase error is the value expressed in degrees (multiplied by 360). The photon follows a straight orbit at the speed of light, and the electron follows suit, while constantly being delayed by the magnetic field which causes the electron to wiggle, thus decreasing its velocity component parallel to the undulator axis. The compact form of equation (10) is usually acknowledged in the literature (Clarke, 2004 ▸),
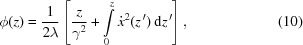
where 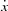 is the *x* component of the normalized electron velocity, 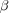 = 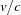, valid in the ultra-relativistic approximation. Even if γ is explicitly present in equation (10), the equation does not depend on the electron energy but only on the magnetic field profile, as expected. It is convenient to explicitly write equation (10) in its final (but less elegant) form used for numerical analysis,
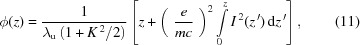
where 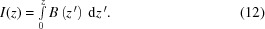
In an ideal undulator, 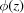 increases by a unit every period. Specifically to a planar undulator, the transversal normalized velocity, 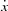, of the electrons is not constant but varies periodically. This also features oscillations in the phase and traditionally limits its analysis to the photons emitted at 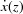 = 0 (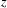 = 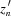), *i.e.* to the photons generated at the smallest bending radius. After evaluating the phase and limiting its domain to the periodic part, the difference between a linear correlation fit and the phase value at 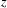 = 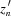 gives the phase errors and its RMS value is used to quantify its spectral quality (Walker, 1993 ▸). As will be described in §4.4, the phase correlates with the local-*K* definition. If the deviation of local-*K* distribution is minimized, the RMS phase error is also minimized.

The electron beam orbit in the undulator is another key parameter which has to be controlled. It has to be measured and optimized as well as used to set the correction magnets during the operation of the FEL. The orbit and the phase error can be optimized only for a given gap but the latter can also be improved with a correction scheme. After evaluating the second field integral, proportional to the orbit, with the equation below, 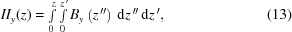
the orbit can still be further optimized with the model in equation (14),
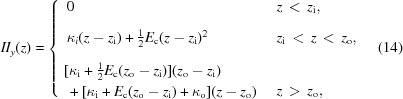
where the entrance (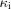) and exit (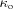) kicks can be evaluated both for the *x*- and *y*-component, while for the Aramis beamline the Earth field (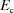) can only be compensated for in the horizontal orbit. The orientation of the undulator and the ambient field (within a small percentage) in the magnetic measurement laboratory is identical to that in the nearby accelerator tunnel. For this reason, no scaling laws have been applied to condition the results of equation (14) before implementing them in the control system of the Aramis beamline.

### 4.4. Optimization algorithms   

#### 4.4.1. Pole height adjustment   

When the undulator is aligned to Bench A, the pole height adjustment, which aims to minimize the RMS phase error, is first carried out. Several methods have been considered and the local-*K* approach (Pflüger *et al.*, 1999 ▸) has been finally retained. This is considered the most robust approach since it does not rely on precise computer modelling and is relatively simple to implement.

The first step is the evaluation of the local-*K* (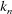) by following the definition of equation (15), where the integral of the main field component, *y*, between two neighbouring zeros, 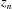, is associated with each pole,
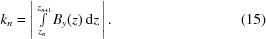
Only the deviation with respect to the average local-*K*, 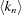, is of any relevance for the optimization process,
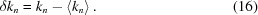
To disentangle the complex relation between the height change in pole *n* and the field change in pole *m*, computer simulations of the field profile variation, 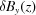, due to a single pole change, 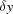, are carried out with *RADIA* (Chubar *et al.*, 1998 ▸) for different gaps (*g*). The local-*K* variation can then be calculated and normalized with respect to 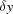 and the following circulant matrix, 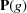, is retained, 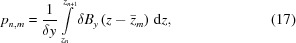
where 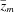 = 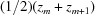. The local-*K* value in equation (16) can be analysed with equation (18) as follows,
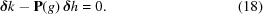
Inverting matrix 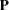 (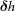 = 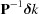) allows for a set of pole height corrections to be evaluated and applied to the undulator magnetic structure. An example is presented in Fig. 14 where several steps of the optimization process are examined with this analysis approach.

#### 4.4.2. Columns height tuning: phase   

**Figure 11:**
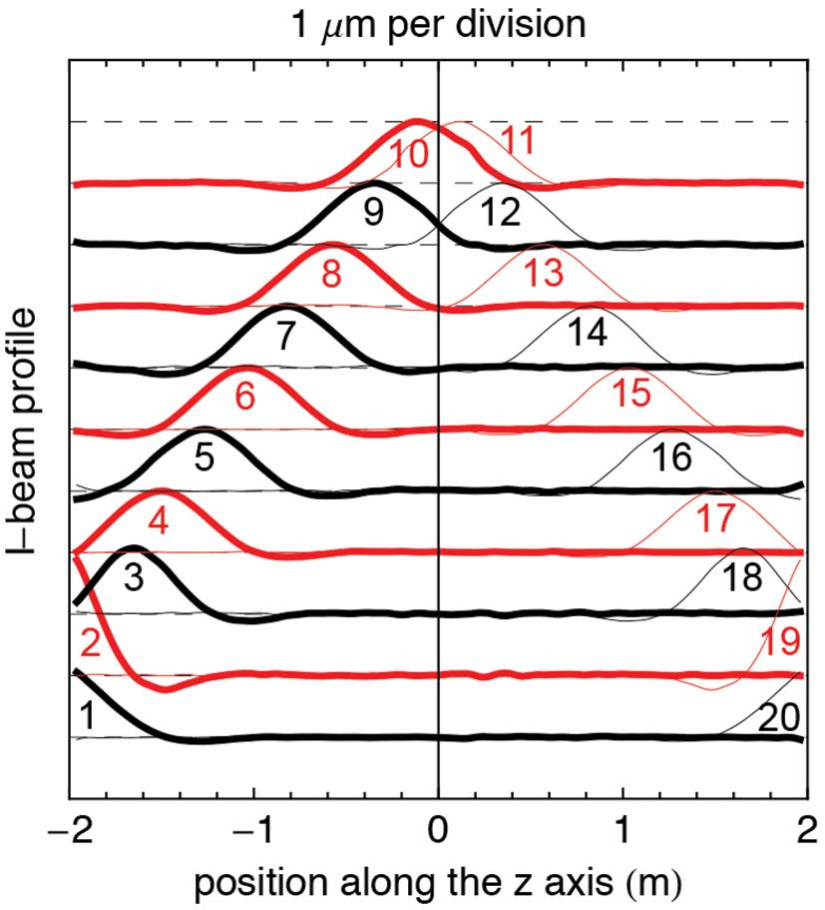
Summary of the magnetic measurement campaign dedicated to the column height studies. Each column, starting from number 1, is elongated by 20 µm. The difference between the magnetic field profile before and after this change is measured and converted to a displacement normalized to 1 µm using the local-*K* algorithm. After the first three columns on both sides, the signature of each column is just the same and has a smooth Gaussian profile. On the contrary, the four columns on the four extremes (upper-left, upper-right, lower-left and lower-right) have an exponential decay due to the lack of a neighbouring column on one side.

A second and very effective tool that is used to optimize the RMS phase error is the adjustment of the column height (see §2). It can be used on a larger range, ±250 µm, and is also accessible after the installation of the vacuum components. In contrast to the pole height adjustment method, changing the column length affects the field profile over about a metre, as shown in Fig. 11 ▸. This has to first be used to minimize the error before the local pole height adjustment, before being used to optimize the field profile after the installation of the vacuum components.

For this analysis, it is convenient to directly use the definition of the phase, as defined in equation (11), and to introduce a small additive perturbation in the field integral, 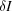. After some algebra and neglecting second-order terms, the phase variation can be expressed with equation (19) below, 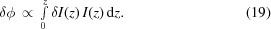
To gain a more intuitive understanding of the phase correction, it is convenient to step back to a simple sinusoidal field profile with a small deviation, 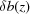, 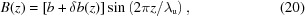
and it is possible to find the intuitive result of equation (21) below,
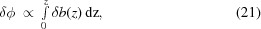
where the phase variation is proportional, in first approximation, to the integral of the field amplitude modulation. In other words, a local increase of the field amplitude introduces an extra delay in the longitudinal electron orbit, thus increasing its phase difference with respect to the photons.

**Figure 12:**
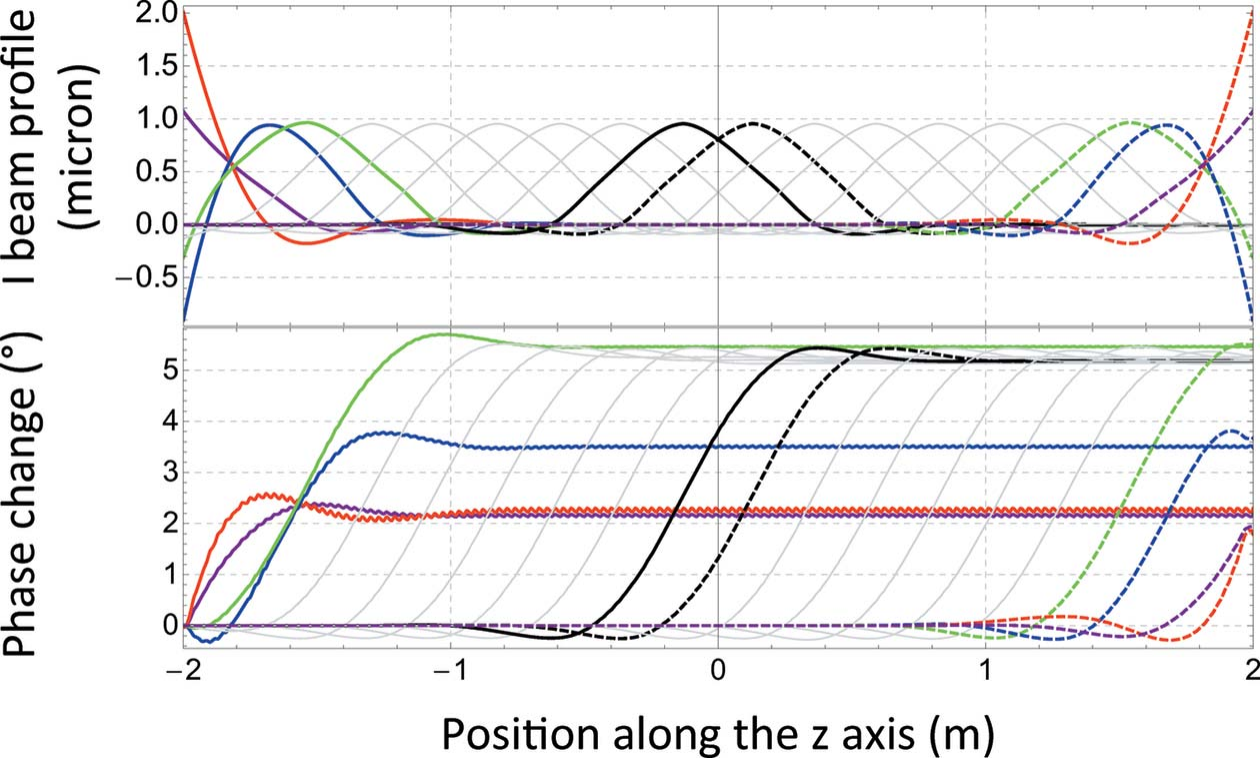
(Top) *ANSYS*© simulations that confirm the measurements results of the column height adjustment method. (Bottom) The corresponding phase change associated with each column adjustment of 1 µm.

**Figure 13:**
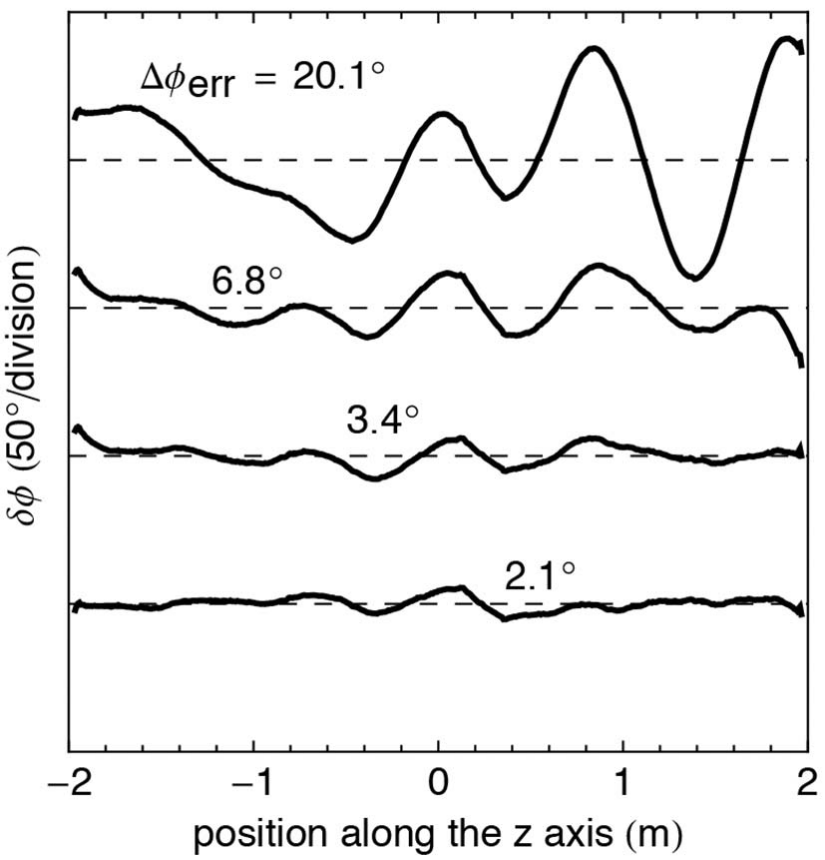
Example of phase optimization using the column height adjustment method. The first curve at the top is the phase error before the optimization (RMS value of 20.1°), while the last curve (RMS value of 2.1°) shows the result after thrice applying the corrections proposed by the algorithm.

The phase optimization first requires an accurate estimation of the deformation of the I-beam produced by the elongation of each column. This is calculated with *ANSYS*© and magnetically measured, see Fig. 11 ▸, to check the FEM model and also to evaluate the correlation between deformation and field modulation, see Fig. 12 ▸. Equation (19) can then be used to convert the field modulation to phase change. Assuming 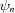 is the phase variation due to the elongation of column *n* of 1 µm, the total phase change can be expressed by the following equation,
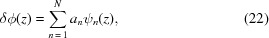
which is valid for small variation (±0.1 mm) within the linear­ity of the mechanics. To minimize the corrections, 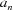, it is also mandatory to constrain them. It is possible to limit them individually, 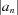 < 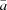. Alternatively, limiting their gradient, which is the maximum variation allowed between two neighbouring columns, 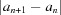 < 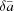, is a better option. This latter is the adopted solution and an example of this optimization is presented in Fig. 13 ▸.

#### 4.4.3. Columns height tuning: axis   

The method developed for the alignment of the undulator (see §4.2) gives the full axis profile. After setting the right pitch and height to minimize the axis profile deviation from a straight line, it is possible to use the columns to further improve its straightness. Using the mechanical model presented in §4.4.2, it is possible to move the upper and lower I-beam to locally displace the centre as needed. Since the position of the columns on the two I-beams is not identical, their optimization must be performed separately, with the use of the following formula,
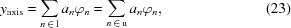
where l and u indicate the index of columns which belong to the lower and upper I-beam, respectively. Since the adjustment is always made by measuring the main field component (at the maximum field of a positive pole) and not the displacement, it is important to correct the sign for the 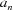 term as follows. If the axis is too low, both I-beams have to move up. However, the upper I-beam has to reduce its field on-axis, while the lower I-beam has to increase its field on-axis. The on-axis 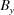 field component should remain unchanged in the ideal case, thus decoupling this optimization from the phase optimization. Unfortunately, the choice of different longitudinal column positions in the two I-beams, which is very important to optimize cost and complexity, breaks this perfect orthogonality. In this specific case, it is not a severe limitation and a fair compromise may still be reached. The axis was initially optimized after alignment and later the priority was given to the optimization of the RMS phase error. This never leads to an axis variation of more than 30 µm.

## 5. Summary of the magnetic measurement campaigns   

The magnetic measurements start with the characterization of the single undulator magnets, continue in industry during different phases of undulator assembly and end in the PSI magnetic measurement laboratories, where the final optimization and characterization are performed before the installation of the undulator in the SwissFEL accelerator. These activities are summarized in this section, using examples to clarify them. The activities are schematically divided into two blocks, mainly the activity performed in industry and the activities carried out in the PSI laboratories.

### 5.1. Magnetic measurements made in industry   

The three momenta of each magnet are measured during their production and only items that are within specification are retained. Before their installation in the block keeper, the magnets are sorted with respect to the horizontal momentum (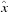 component) to minimize the associated vertical orbit. This is a priority since no simple system is designed to adjust the vertical orbit within the undulator assembly.

After this exercise, the field integrals of each block keeper are then measured. The value of the 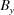 integral is used to centre the measurement, due to its highly symmetric profile in the *x* direction. The centre value, 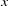 = 0, of the 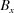 integral is used to position the blocks along the undulator length to further minimize the vertical orbit deviation.

When the magnets are installed in the I-beam, the magnetic field is then measured. The analysis of the main vertical component is used to adjust the position of the blocks. With these magnetic data, it is possible to assess the longitudinal position, *z*, of each magnetic pole and improve the position of every block to recover a better periodicity. By looking at the magnitude of the field around a pole and applying the technique of the local-*K*, as specified in §4.4.1, it is possible to recover the systematic height error amongst the blocks with the help of non-magnetic steel strips below the block. If a large height variation is observed within a block, it is disassembled and inspected before the magnets are removed and installed in another block keeper. These checks are very effective in preventing the later discovery of a large magnetic error in the PSI laboratories, where the disassembly of the entire undulator would take more time and more manpower.

The final procedure that is performed in industry is the vacuum testing. All the individual components are cleaned in an ultrasonic bath to enable them to be compatible with an ultra-high-vacuum (UHV) environment and are then assembled in clean rooms. Nevertheless, at this stage, it is still possible to have contaminants and therefore the final assembly is tested in a dummy chamber that has been previously cleaned and tested. If the expected vacuum level is achieved, the assembly is shipped to the PSI laboratories.

### 5.2. Magnetic measurements made at the PSI laboratories   

#### 5.2.1. Undulator alignment and optimization   

**Figure 14:**
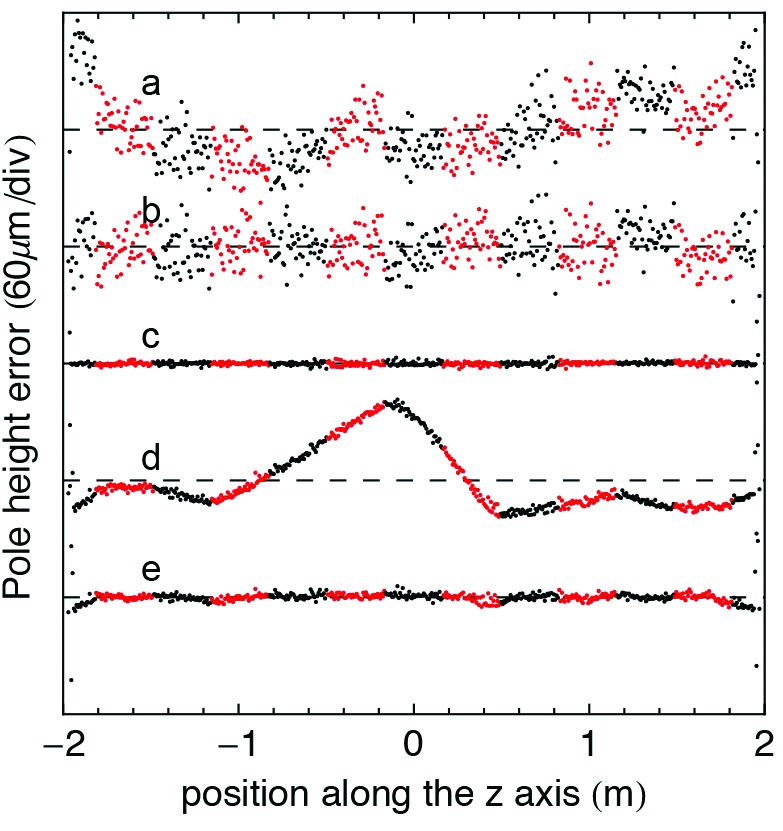
An example of five steps of the optimization procedure using the local-*K* analysis to illustrate the results is presented. (*a*) Profile of a pre-optimization magnetic structure after alignment on Bench A. (*b*) After column height adjustment. (*c*) After pole height adjustment. (*d*) First measurement after alignment on Bench B. The magnetic structure has meanwhile been disassembled and reassembled within the vacuum chamber. (*e*) After column height adjustment. The red and the black dots represent poles belonging to different block keepers.

Each undulator is finally assembled and magnetically tested at PSI. The magnetic arrays arrive at PSI in a separate parcel which is then assembled into its final support frame. This is a complex operation and requires trained personnel and some days of work. The first magnetic measurements are made without the vacuum chamber on Bench A. After the alignment (see §4.2) and the axis optimization (see §4.4.3), the magnetic field is measured at a gap of 3.8 mm, which corresponds to a *K*-value of about 1.4. This value was chosen because it is in the middle of the operating range, *i.e.* between 1.0 and 1.8. The optimization based on the column height adjustment is first used to minimize the RMS phase error. This is mandatory to enable the application of the local pole height adjustment, which is limited to the narrow range of only ±30 µm. An example is given in Fig. 14 ▸ where the local-*K* analysis is used to illustrate this procedure.

After optimization, the field is measured over the full operational *K*-range to check if the RMS phase error is within the tolerance and to verify that, at the minimum gap of 3.0 mm, the *K* value is above 1.8. The former was always verified, while the latter was not achieved for all undulators. This was not due to a lack of magnetic strength but due to the unreliable setting of the encoder offsets, set during the assembly of the frame when the upper and the lower units were still apart. Considering the fact that a change in the offset could lead to a potential hazardous operation where an error could cause severe damage to the structure, it was preferred to change the length of the columns uniformly to meet this requirement.

#### 5.2.2. Undulator final optimization   

The second test campaign is performed with the undulator in its final configuration. The upper and the lower magnetic arrays are fixed mechanically together before removing the columns. They are first unscrewed from the upper outer I-beam, fixed though the inner I-beams to a sliding table, unscrewed from the bottom outer I-beam and finally removed from the support frame. The columns are then removed and the inner I-beam slid inside the vacuum chamber. The columns are set back in the I-beams through a set of small flanges in the vacuum chamber and the bellows fixed together with the rest of the vacuum components: ion pumps, gauges, thermocouples *etc*.

Similarly to the procedure previously described for Bench A, the undulator is then positioned and aligned on Bench B. The first measurement results would negatively impress because of the poor quality of the phase after the previously described manipulation. Its value regularly exceeds 50°, which might induce doubts about the relevance of the previous optimization. This is clearly not the case as illustrated in the example in Fig. 14 ▸, where the local-*K* profile is detailed. If the phase is varying substantially, the difference in the local-*K* between two neighbouring poles is very small. This is a fundamental result which confirms that the local pole height correction is still present even after the disassembling and reassembling of the structure and the measured effect is only related to the manipulation of the columns which clearly change their height in the range of ∼10 µm during this activity. Therefore, to reduce the phase error it is required to apply an additional column height optimization and the phase of all the undulators in the series can be set below 3° at the optimum gap of 3.8 mm. Comparing this result with the value achieved after the first optimization, where usually the phase was reduced to about 1°, nevertheless should trigger discussion about the possibility of applying the pole height adjustment within the vacuum chamber. This would improve the phase result while substantially reducing the optimization time and the required resources. A working solution is not yet available for implementation but a project is ongoing at PSI to release the first version of such a system by the end of 2018.

#### 5.2.3. Undulator magnetic characterization   

**Figure 15:**
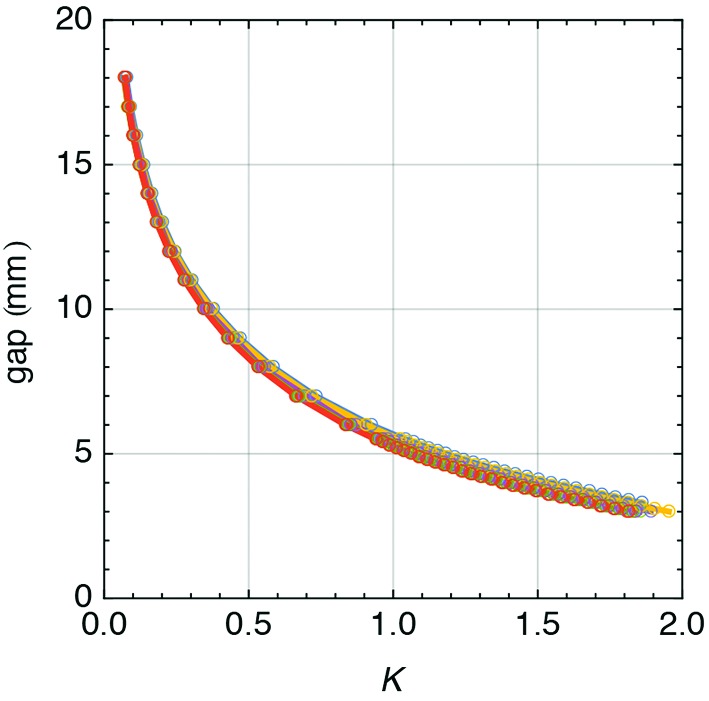
Measurements results of the gap *versus* *K* correlation for 40 different gaps and all undulator modules.

**Figure 16:**
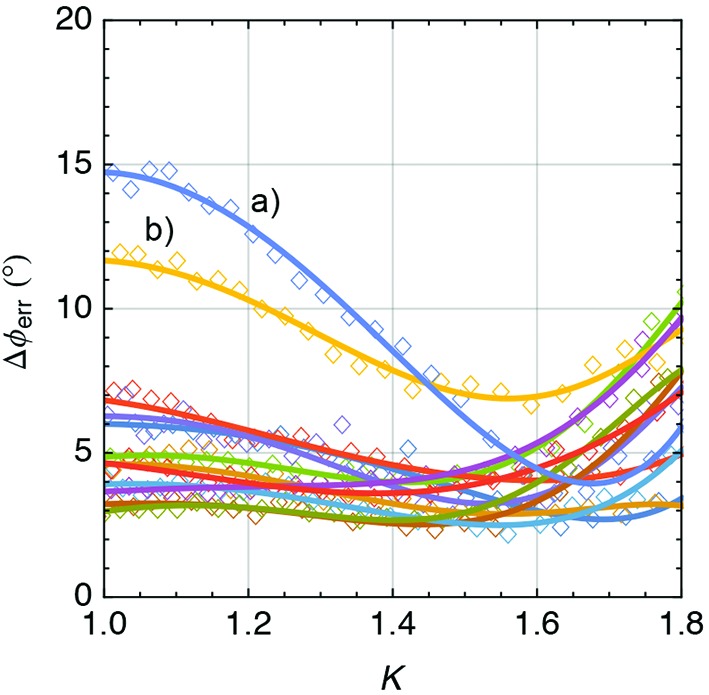
Summary of the RMS phase error for all undulators as a function of *K*. They are all within the specifications (<10°) but two (*a* and *b*). Undulator (*a*) had a problem with the mechanical stability of the gap drive system while undulator (*b*) was equipped with magnets with higher magnetic errors.

After the second optimization campaign, all undulators undergo a full magnetic characterization which lasts for around 12 h. This is fully automated and can run without human supervision, during nights and weekends. During this campaign, the on-axis field is measured for all gaps, see Fig. 15 ▸, from fully open at 18.0 mm to fully closed at 3.0 mm, with a total of 40 measurements. Fig. 16 ▸ shows a summary of the RMS phase error as a function of *K* > 1 for all undulators. Its value is lower than 10° for all but two undulators. A more detailed study showed that in one case the problem was related to the magnet quality (different magnet supplier) and a second to the mechanical stability in the range of a few micrometres. In the latter, the issue could be in the gap drive system but other mechanical components could also produce similar behaviour. There was no time for further investigation and the undulator was accepted and installed in the Aramis beamline.

The required parameters for the Aramis operation are extracted out of this magnetic measurement campaign: the *K*-value, the entrance and the exit kicks in both the vertical and horizontal plane as well as the earth field vertical component. Moreover these data contain fundamental information also for the setting of the phase shifter as will be detailed in §6.3.

#### 5.2.4. Positioning of the alignment quadrupoles   

For the beam-based alignment of the undulators, a set of fixed permanent-magnet quadrupoles (called alignment quadrupoles, Qals) has been implemented, partially following the strategy used at the LCLS (Nuhn *et al.*, 2006 ▸; Nuhn, 2009 ▸). Two Qals are located at both ends of the magnetic array and are pre-aligned to the magnetic axis in the laboratory. At the end of the characterization campaign on Bench B, the Hall probe is well positioned along the final undulator axis and can be used to precisely assemble the Qals. The procedure at this stage is fairly simple. The probe is moved along the undulator axis where the Qal is to be inserted. The reading of the probe is then recorded and used as a zero reference value. The Qal is then installed and moved in the transversal plane (*xy*-plane) until the reference reading is recovered in both axes. The Qals are mounted on a guiding system and are moved out of the beamline during regular operation. A reproducibility study has been carried out to understand the errors introduced during the displacement of the quadrupoles in and out of the beamline. An error of a few micrometres has been measured, which is within the requirements.

#### 5.2.5. Transfer function measurements   

**Figure 17:**
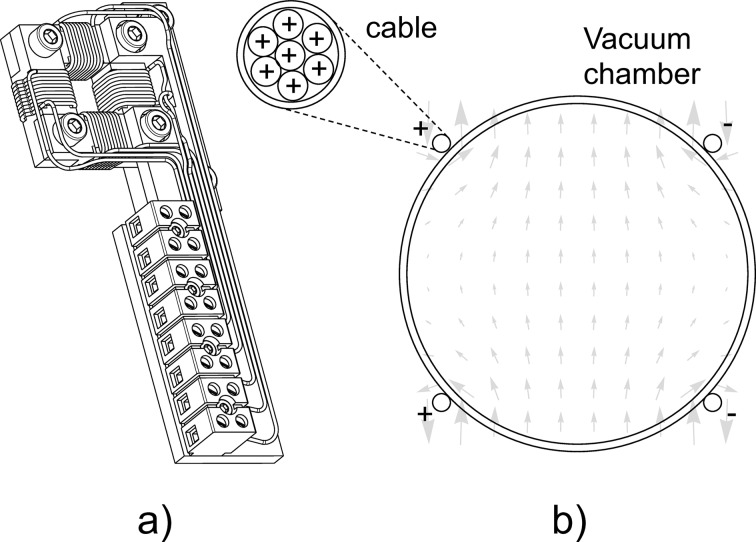
Orbit correct magnets. (*a*) The window frame dipoles used for the correction of the horizontal and vertical orbit kicks, used both at the entrance and at the exit of the undulator. (*b*) The Earth field corrector coil made of a multi-conductor cable wrapped over the vacuum chamber to produce a uniform dipole magnetic field all along the undulator axis.

To effectively improve the orbit with the correction scheme (14), the transfer functions of the corrector magnets have to be measured. Window frames with vertical and horizontal dipoles (WFDs) used for the entrance and exit kick corrections, seen in Fig. 17(*a*) ▸, are measured in the PSI magnet laboratory. The correlation between field and current is linear and very reproducible between the two axes and among different units and no hysteresis has been measured in the range of interest.

**Figure 18:**
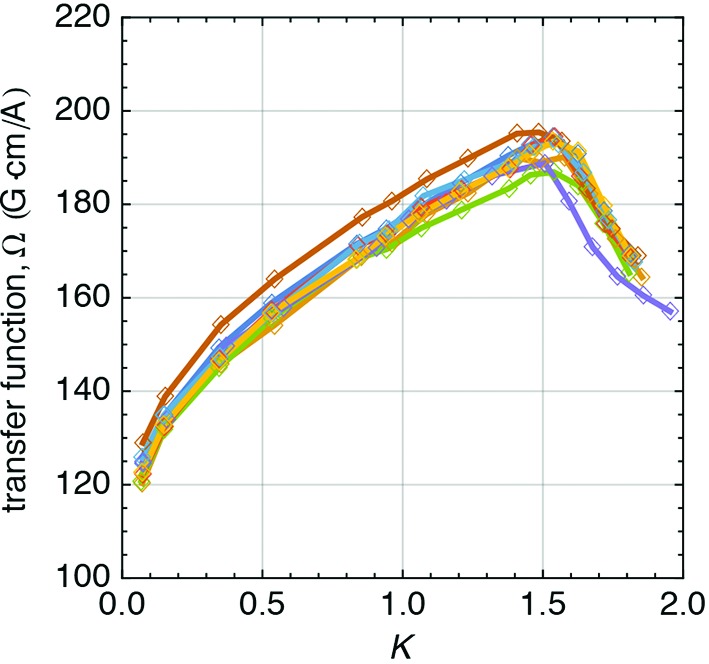
Transfer function of the long coils as a function of the undulator *K* value, measured with the moving wire system.

On the contrary, the Earth field correction coil [long dipole (LD), *i.e.* long coil] cannot be measured independently because it is assembled on the undulator vacuum chamber, see Fig. 17(*b*) ▸, and its magnetic field is strongly coupled with the undulator’s iron poles. Its transfer function is deduced through measurements made on Bench B with the moving wire system. While the correlation between field and current is constant, due to the low field excitation, the transfer function depends on the undulator *K* (see Fig. 18 ▸). As the undulator gap decreases, the value of the transfer function is seen to increase, as expected by the magnetic coupling, until the poles saturate. At this point, the transfer function reaches a maximum and then it decreases. The magnetization status of the poles is not defined by the corrector (which generates a very low field of a few Gauss) but by the field in the undulator (*K*) and it increases with decreasing/closing gap.

#### 5.2.6. Phase shifter measurements   

**Figure 19:**
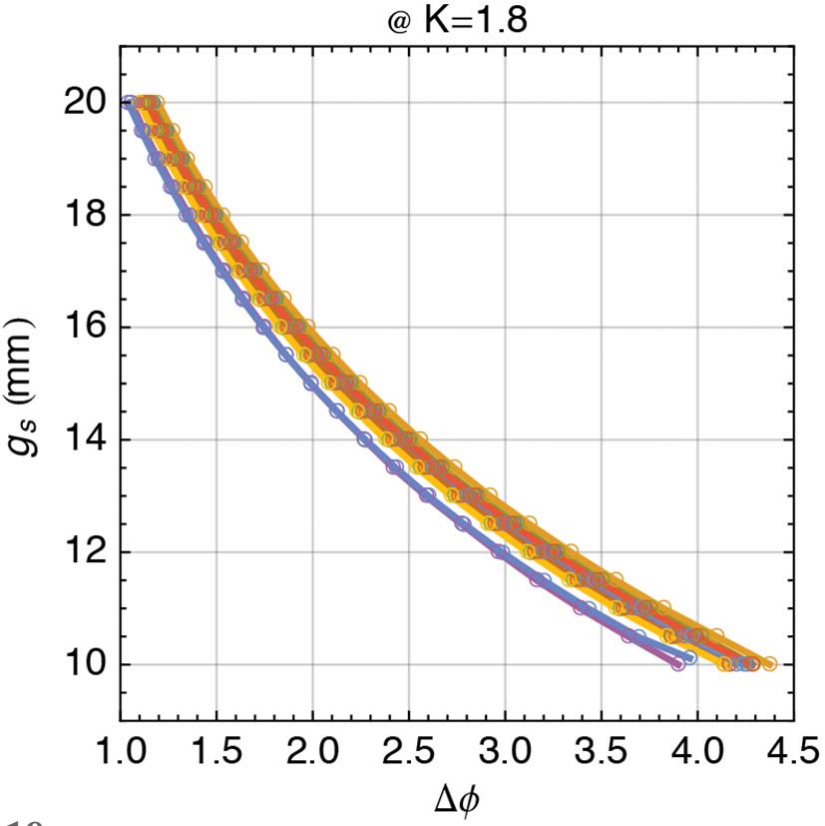
Gap (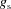) *versus* phase (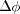) relation, measured for all phase shifters. The results are presented for *K* = 1.8 which is the design value and the worst-case scenario for the phase shifter strength.

The phase shifters are measured, shimmed and characterized at PSI. The overall procedure is similar to the one discussed for the U15 series with few differences. The optimization parameter is the electron orbit, while the operation parameter is the phase. The former has a definition almost identical to the one of equation (11), differing only in the missing linear term in *z*, which is correlated to the drift, as seen in equation (24), 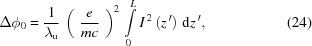
where *L* is the length of the phase shifter, *I* is its field integral and the subscript zero is a reminder that 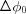 is calculated for an undulator *K* = 0. It is more convenient to calculate the action of the phase shifter on top of a pre-existing drift section, *i.e.* to separate the two contributions, as will be evident in §6.3. Equation (24) can be extended to the generic *K*-value of the undulator, where it is instructive to highlight that 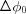 depends only on the phase shifter gap (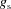), 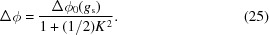
Finally, 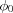 is measured and the results are shown in Fig. 19 ▸ in the form of equation (25) normalized with *K* = 1.8 and presented as its inverse function.

## 6. Summary of the magnetic modelling campaign   

All the parameters that have been described in the previous sections that are relevant for FEL operation must be modelled and implemented in the EPICS control system (see §7). This section will provide a description of all the models that were developed for the Aramis beamline, to simplify its operation and to improve the overall performance of the FEL process.

The models are preferentially derived as functions of undulator *K*-value instead of the more traditional gap value because this latter parameter is directly related to the photon energy through the undulator fundamental equation (8). Nevertheless, it is also important within a feedback loop to also derive the inverse function, as will become more clear later in the section, to estimate the *K*-value for a given readout gap value in the encoders.

Each undulator module has been measured individually and this information has been used to build individual models to achieve the highest possible accuracy for the FEL operation.

### 6.1. Gap *versus* *K*   

The relationship between the gap and the magnetic field of a hybrid structure has been predicted through numerous studies (Moog *et al.*, 2017 ▸). Taking into account the non-linear behaviour of the magnetic steel poles, the general form of the widely used result for the magnetic field amplitude is shown in equation (26), scaled for *K*,
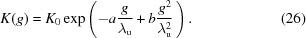
Equation (26) can and should be generalized to include higher-order polynomials to cater for the high accuracy required for the FEL operation.

Nevertheless, modelling *K* *versus* gap is not the natural way for setting up the undulator beamline. After calculating *K* for the specific operational needs, the gap of the undulators have to be set and this requires the inversion of equation (26). This is done numerically and requires more resources of the control system. To overcome this inconvenience it is possible to model directly gap *versus* *K*. As long as *K* *versus* gap has an exponential nature, the natural choice for its inverse is to use a logarithm. Using equation (27) below, 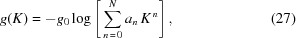
with *N* = 3 gives already good results with maximum relative deviation of about 0.3%; the fit results are presented in Table 3 ▸. Restricting the domain to *K* > 0.5 reduces the errors below 0.1%. The details of the fitting procedure is reported in Appendix *B* [App appb].

### 6.2. Orbit corrections   

The residual field errors of the undulator introduce distortions in the electrons orbit. They have been measured and parametrized with equation (14), which is designed for the specific SwissFEL correction strategy. The entrance and exit kicks (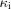 and 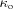) for both the vertical and horizontal planes are tabulated as a function of discrete *K* and fitted with a polynomial function of order 7. The same is done for the Earth field correction, 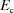, but only for the horizontal plane because there is no simple means to correct it on the vertical plane in an hybrid undulator. As already discussed in detail in §5, the WFDs and the LD magnets are measured at PSI. The WFDs are designed to correct the entrance and exit kicks, κ. They have a linear correlation between the dipole field and the current in both planes and it is estimated to be 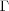 ≃ 23.3 G cm. The equation to control their current reads
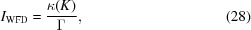
and it is valid for the entrance and exit kicks as well as for the vertical and horizontal plane. The LD magnets are designed to correct the Earth field, 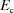. Like the WFD, they have a linear correlation between field and current but the correlation changes when the undulator *K* changes. With minor modifications, it is possible to adapt equation (28) to the new functionality 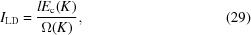
where 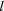 is the length of the LD coil and Ω is its transfer function shown in Fig. 18 ▸, modelled using a seventh-order polynomial function.

The accuracy of this correction strategy was determined by feeding the fits for 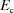 and κ at each value of *K* back to the piecewise model in equation (14) and subtracting it from the measured orbit. The resultants are an indication of the processing and modelling error, which are acceptable up to a standard deviation of about 2 µm for an electron beam energy of 5.8 GeV. Table 4 ▸ shows the maximum values of standard deviation for each undulator across the whole range of *K* before and after applying the correction strategy.

### 6.3. Phase matching   

The electric field produced by the wiggling electrons is periodic within each undulator (neglecting the small phase error). However, the phase of the photon emissions generated by two respective neighbouring modules can be mismatched. For undulators with a fixed magnetic field, it is enough to position them correctly, with the right distance between each module, to guarantee that the emissions are always in phase with each other. Moreover, changing the electron beam energy does not affect the relative phase among different undulator modules, as was deduced from equation (11).

**Figure 20:**
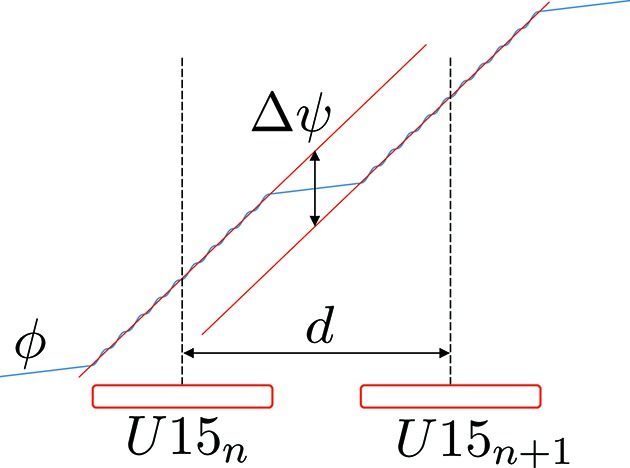
Schematic view of the phase increase, ϕ, inside and between two neighbouring undulators. The fundamental parameters 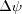 and *d* are highlighted, where the former (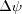) is the offset between the linear extrapolated phase between two undulators (the slope of the two red lines has to be identical and equal to 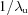 if *K* of both undulators is the same) and the latter (*d*) is the distance between the central zeros of the two consecutive undulators.

On the contrary, for variable-gap undulators, the phase condition changes for different *K* (Li & Pflueger, 2017 ▸). A simple model can be deduced by considering the periodic field of the undulator. Inside the undulator, the phase increases by definition of a unit per period. Neglecting the oscillations inside a period, the phase along the beam axis increases linearly as expressed by equation (30) below, 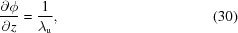
while in the ∼0.75 m space present between undulators (in the drift part) the rate of change of the phase varies at a slower rate, 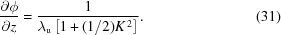
This occurs because the longitudinal velocity of the electrons increases upon exiting the undulator, due to the absence of a magnetic field. However, the distance between the electron and the photon still increases outside the undulator but at a lesser rate (see Fig. 20 ▸). In this model, the phase increase between two modules is
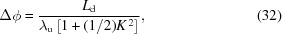
where 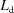 is the distance between the two modules. Introducing an additional delay, 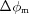, the matching condition can be expressed with the following equation, 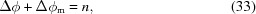
where *n* is a positive integer. To solve equation (33), it is convenient to introduce the matching function *M*, closely related to the modulo 1 function, which is defined using the ceiling function, 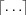, in equation (34),
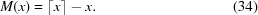

*M* is a periodic function of period 1 and monotonically (linearly) decreases within a period. Therefore, the phase matching equation (33) can be solved in the following manner, which is simple to calculate,
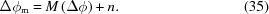
Equation (35) is more general and can be used even when the simple model previously described in equation (32) is no longer valid and the undulator’s end fields are correctly taken into account. Therefore it is convenient to modify it and to highlight the experimental parameters that can be measured to correctly take into account the entire field profile of an undulator, including the end field and the stray field. In this approach, two parameters are sufficient to estimate the additional phase required for the matching condition. These are the distance, *d*, between the central magnetic field zero (it is an antisymmetric profile with a zero field value in the middle) of two neighbouring undulators and the extrapolated phase difference between them, 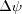: see Fig. 20 ▸, where these two parameters can be easily identified. The new and equivalent condition for phase matching reads as follows,
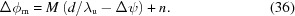
The laser tracker measures the position of the undulators in the tunnel with an accuracy of ±20 µm. This information is used to estimate the distance, *d*, between the magnetic centres of two neighbouring undulators. This does not come without additional uncertainty, because the magnetic array position may vary with respect to that of the I-beam. However, this is nevertheless the best guess available at the early stage of assembly.

To estimate 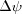 it is essential to use the full-field profile of two neighbouring undulators to correctly take into account the details of the end fields. To prepare the data, the two undulator magnetic profiles have to be scaled to a given *K*-value. This is done by the linear interpolation of two measurements around the target *K*. Finally, the new field profile, 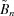, is defined as follows, 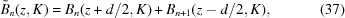
where 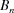 is the vertical field of the *n*th undulator and 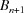 is that of its neighbour downstream, assuming both are centred originally around their middle zero field value. The phase increase of this magnetic system is then calculated with equation (11) and 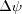 can be calculated as already presented in Fig. 20 ▸, *i.e.* evaluating the difference between the linear fit of the phase increase of the first and second undulator, respectively.

**Figure 21:**
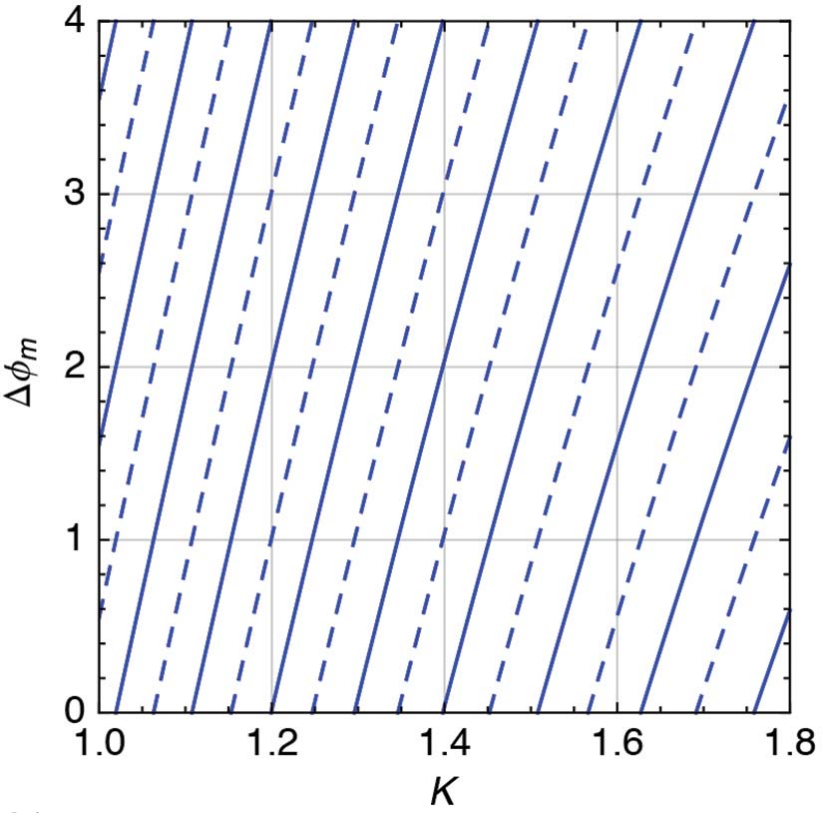
Example of phase (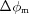) *versus* *K* for a pair of undulators. There are multiple solutions for a given *K* as is naturally expected by a period function like the phase.

**Figure 22:**
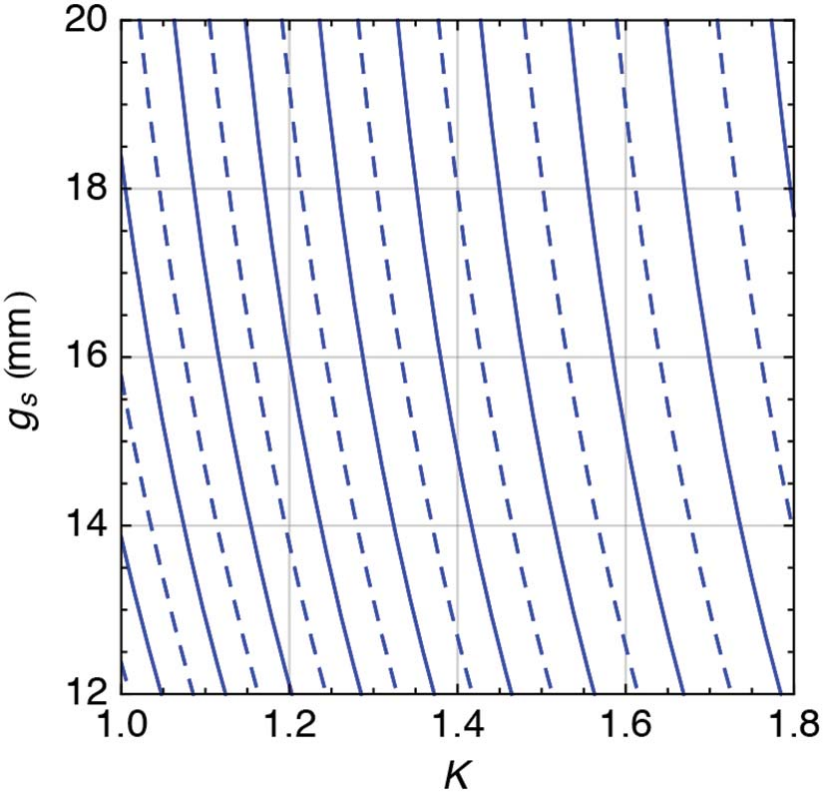
Example of gap (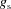) *versus* *K* for a given phase shifter and a pair of undulators. The phase shifter can be operated between 12.0 mm and 20.0 mm, and in the worst case of *K* = 1.8 there are still two solutions available.

After calculating 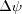 for different *K*, it is modelled using the simplified model described before in equation (32), 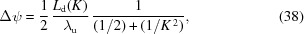
where 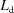 is used as a fitting parameter. An additional *K* dependence is added to take into account the different scaling of the matching end field with respect to the periodic field, resulting in an equivalent different length, 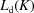. A second-order polynomial function achieves acceptable results, compatible with few degrees of additional errors. In Fig. 21 ▸ an example of equation (36) is presented as a function of *K*. Equating 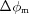 to the right-hand side of equation (25), 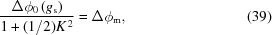
it is possible to calculate the gap of the phase shifter to fulfil the matching condition. Inverting the measured function 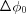 gives an estimation of 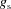, 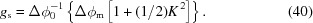
An example of equation (40) is presented in Fig. 22 ▸ where multiple solutions are present for a given *K* as expected from the periodic nature of the phase. Nevertheless, the number of solutions that are available for the limited phase shifter gap range decreases with increasing *K*. This last result confirms the intuitive idea that a phase shifter has to be designed with respect to the largest *K* available at the specific beamline.

Having a simple physical model for the phase matching is also an advantage in the later stages of the FEL operation. The additional information gained with the operational experience can be later included in this model. The new data can be fitted with the previously introduced equations, which have the advantage of having a clear physical interpretation.

## 7. SUBLIME   

The precise operation of the U15 undulators can be ensured through the implementation of a consistent and reliable system of models, presented in §6, which are derived from data obtained during the measurement campaign discussed in §5. This system of models, which is collectively referred to as SUBLIME (aramiS Undulator BeamLIne ModEl), is made up of several individual fits, each of which is used to accurately calculate the parameters that are essential for undulator operation.

The aim of SUBLIME is to produce individual values for the undulator, corrector and phase shifter parameters that need to be set for operation, based on the common value of the deflection parameter, *K*, that corresponds to a specific user-defined radiation wavelength. Once this wavelength is specified, a value of *K* can in turn be calculated accordingly for a given electron beam energy value, through the resonance equation expressed in equation (8). The *K* value is then passed on to SUBLIME from the control room to calculate all the parameters necessary for operation.

**Figure 23:**
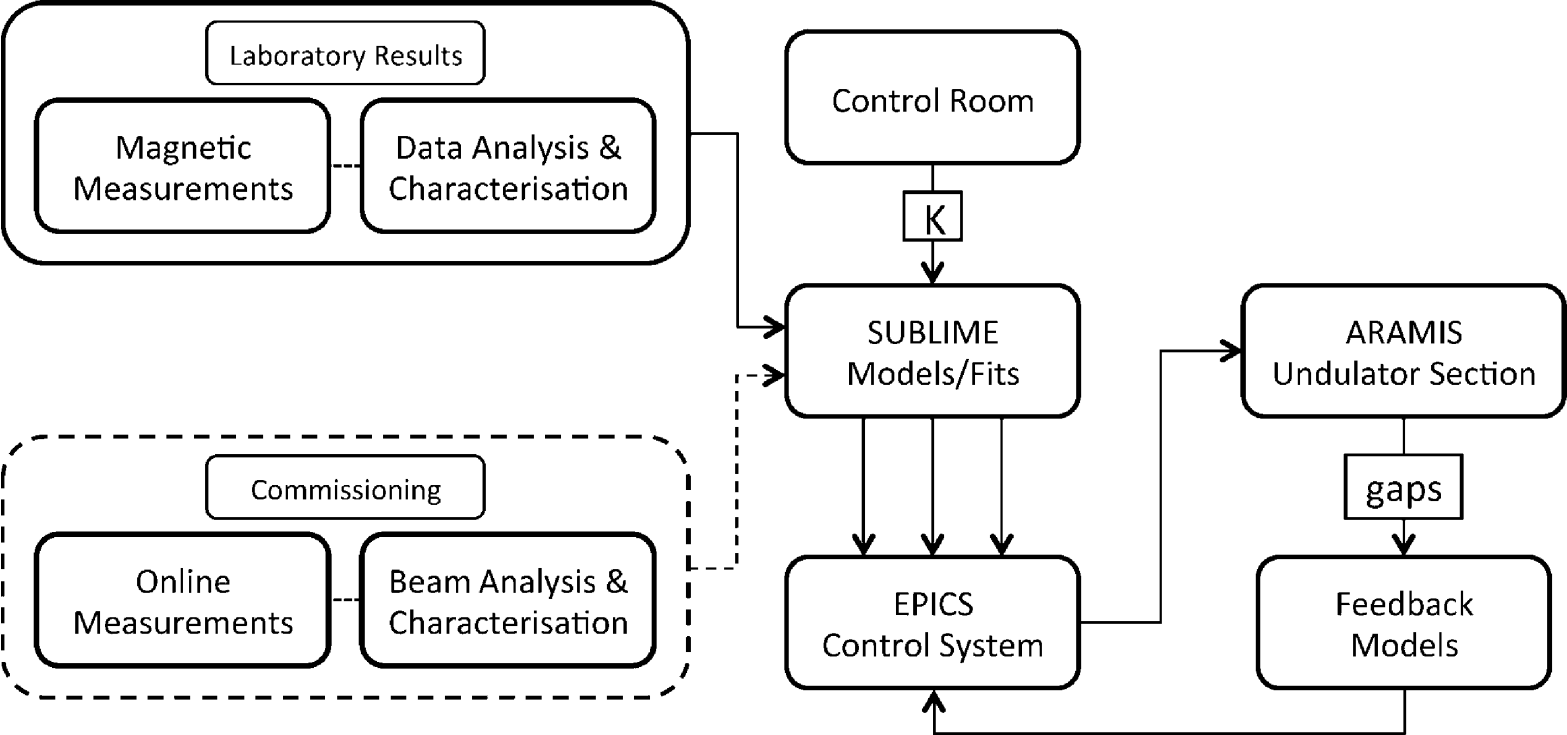
System summary for Aramis beamline operation. The *K*-value is obtained from the user-defined value of radiation wavelength in the Control Room and is then passed on to the SUBLIME models, where values for the parameters that control the undulators are produced (undulator and phase shifter gaps and orbit corrector currents) and passed on to EPICS. The SUBLIME models are all derived from the magnetic measurements of all U15 undulators and their respective correctors. These parameters are then implemented on the undulator line through EPICS. Amendments may still need to be implemented on SUBLIME once commissioning of the Aramis beamline is underway and online measurements are available. The value of the undulator gap is continuously acquired from each U15 undulator while setting the undulator gap for operation, and is used to calculate the current that needs to be applied to all orbit correctors in order to prevent loss of electron orbit while setting the undulator gap. Feedback models are used for this purpose.

**Figure 24:**
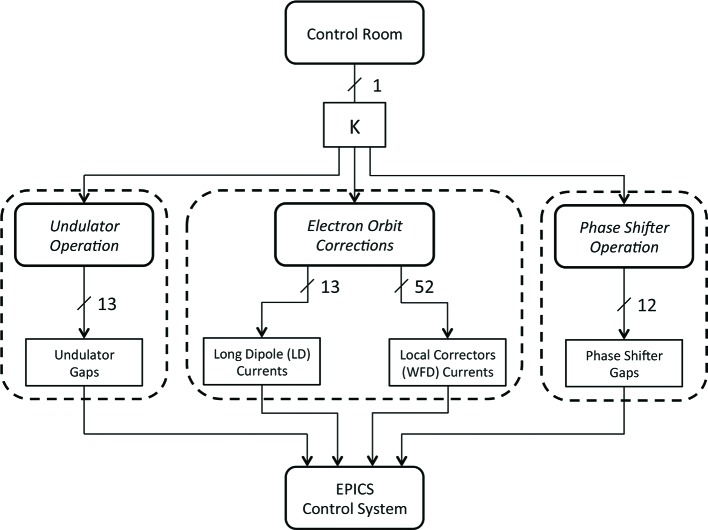
Overview of the SUBLIME system of models: 13 undulator gap values are provided to achieve the desired radiation wavelength when the electron beam moves through the undulators, 13 and 52 current values are applied to the long dipole coils (LD) and the local correctors (WFD), respectively, to correct the electron orbit, and 12 phase shifter gap values are applied to the phase shifters that result in the matching of the radiation produced by two consecutive undulators. All values are calculated from a single input value of *K* obtained from the control room.

These values are subsequently fed to the EPICS control system to ultimately obtain radiation with the desired wavelength. A block diagram of the system in a holistic context may be seen in Fig. 23 ▸. The SUBLIME block returns three main sets of parameters that are necessary for undulator operation. These parameters are shown in Fig. 24 ▸ and include the setting of the undulator gap, the orbit corrections and the phase shifter gap.

While the equations are common for all undulator gaps, electron orbit correctors and phase shifters, the coefficients making up the models were individually produced for each unit by fitting the model to the magnetic measurements that were recorded for each individual component. This was possible since the undulators, long coils, local correctors and phase shifters were individually characterized during the measurement campaign.

The implementation of individual models for each component was possible since each undulator, corrector and phase shifter is individually controlled in the beamline during operation. Separate gap-drive systems are dedicated for all undulator and phase shifter gap adjustments, while separate power supplies are dedicated for individual current settings for the LCs and WFDs, respectively. The block diagram in Fig. 24 ▸ represents the SUBLIME system of models in more detail.

## 8. Conclusions   

The U15 series had several innovative elements which could have introduced additional uncertainties during the magnetic assessment. The closed frame obliged the undulator to be shimmed for the first time based on SAFALI measurements, whereas, in previous projects, SAFALI had only been used for the characterization in the vacuum chamber. This was proven to be very successful and could also be adapted to the requirements of a series production. The automatization of the shimming improved the quality and reduced the time and the manpower required. The alignment procedure and the optimization of the undulator axis also have to be acknowledged as a new and effective tool which reduces the time and improves the reliability of the entire process. The column height adjustment, with pre-calculated correction for each column in a single step, substantially reduced the time required and limited the hazardous manipulation of those critical components.

All the knowledge of the magnetic properties of the Aramis beamline has been summarized in the SUBLIME model. The approach developed for the phase matching integrates all the properties of the undulators, including the accurate referencing in the accelerator tunnel, and the magnetic characterization of the phase shifter magnetic properties.

The magnetic assessment of the first U15 prototype was crucial to introduce few but important improvements in the design.

The column’s layout was modified to give room to a second counter-nut, not present in the first assembly of the prototype. This increases the mechanical stability of this crucial component, avoiding small but measurable relative displacement of the two parts of the column. The RMS phase error was increasing above the specified value of 10° after cycling the gap between open (18.0 mm) and closed (3.0 mm) more than 100 times. This was no longer observed after the modification of the column design.

A water-cooled plate was introduced to stabilize the temperature of the servo-motors implemented in the gap drive system. During the regular operation, the gap is set once and the undulator is operated for time ranges of several minutes to a few hours at the same strength with the motors off. However, there are experiments where the photon energy (*i.e.* the undulator strength, *K*) has to be changed continuously and the servo motors have to stay on for about an hour. The heat produced during this operation was flowing from the motor to the wedge and consequently changing its size enough to compromise the RMS phase error as well as the measured relation between *K* and gap. Difficult to simulate, it was easy to measure magnetically and prove that the cooling system was mitigating this effect.

**Figure 25:**
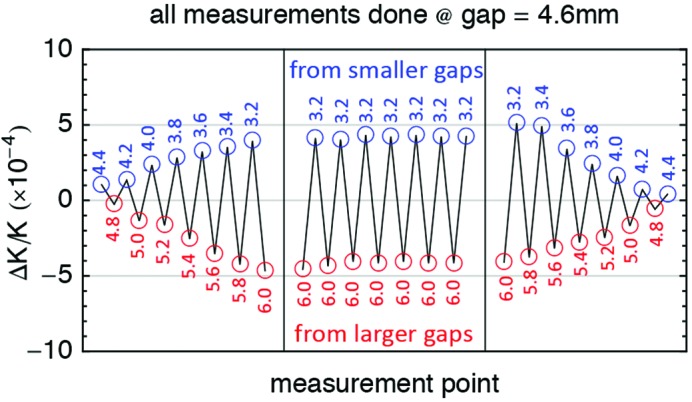
Example of hysteresis measurements made at a gap of 4.6 mm. The red markers indicate measurement points made after setting the gap to a value larger than 4.6 mm and then closing to 4.6 mm. The blue markers indicate measurements made after setting the gap to a value smaller than 4.6 mm and then opening to 4.6 mm.

A magnetic hysteresis was measured on the prototype, *i.e.* the relation between gap and *K* depended on the history. If the gap was set starting from 18.0 mm, it was seen to be different than if it was set starting from 3.0 mm (see Fig. 25 ▸). For this reason a new design of the block keeper was made, to increase its mechanical stability and in the meantime to optimize its magnetic design. Conversely there was no impact on the hysteresis measurements: their effect is still present in the U15 series and it must be due to a different source of uncertainty. The hysteresis has to be taken into account during the operation of the U15 undulators and the gap has always to be set starting from larger to smaller gap (*i.e.* in the closing direction). When changing direction, the gap has to be open above 10 mm before closing it at the new set value.

## Figures and Tables

**Table 1:** SwissFEL Aramis beamline design parameters

Electron accelerator	Beam energy	2.1–5.8 GeV
	Energy spread (r.m.s.)	350 keV
	Normalized emittance	430 nm
	Peak current	3.0 kA

Undulator parameters	Undulator period, λ_u_	15 mm
	*K*-value range	0.1–1.8
	Active length	48 m
	Total length	60 m

Photon parameters	Wavelength	1–7 Å
	Pulse energy	0.01–1 mJ
	Bandwidth	0.04–3%

**Table 2:** Summary of the reproducibility study performed on the two magnetic measurement benches, A and B

	Δ*K*/*K* × 10^−4^		Δϕ_err_	
	Short term	Long term	Short term	Long term
Bench A	0.36	2.15	0.72	0.66
Bench B	0.32	0.65	0.24	0.21

**Table 3:** Coefficients of the cubic logarithmic model for *K* > 0

Undulator	*g*_0_	*a*_0_	*a*_1_	*a*_2_	*a*_3_
U35159	4.89055	−0.00373	0.40899	−0.07027	0.00208
U38764	4.87679	−0.00324	0.37255	−0.06130	0.00402
U40730	4.74887	−0.00370	0.36901	−0.04653	−0.00025
U42718	4.59281	−0.00527	0.35675	−0.02844	−0.00505
U42292	4.51221	−0.00594	0.34394	−0.01734	−0.00846
U40046	4.54499	−0.00573	0.34862	−0.02130	−0.00721
U40101	4.94700	−0.00258	0.40970	−0.07252	0.00521
U40971	4.64033	−0.00530	0.36492	−0.03504	−0.00360
U42287	4.65707	−0.00403	0.36204	−0.03387	−0.00386
U41020	4.82170	−0.00423	0.39408	−0.06092	0.00324
U40679	4.99419	−0.00099	0.41996	−0.07730	0.00673
U41694	4.84378	−0.00254	0.37214	−0.05944	0.00439
U41802	4.71818	−0.00528	0.37755	−0.05342	0.00292

**Table 4:** Effectiveness of the orbit correction model on a 5.8 GeV e-beam

	H-orbit (µm)		V-orbit (µm)	
Undulator	Before	After	Before	After
U41802	10.80	1.76	1.61	0.83
U41694	14.95	1.41	3.55	1.84
U40679	6.59	1.35	2.29	2.27
U41020	15.31	2.37	5.44	1.97
U42287	9.82	1.57	4.13	1.32
U40971	6.77	1.40	4.92	1.01
U40101	8.65	2.64	5.32	3.07
U40046	10.41	1.29	5.48	1.45
U42292	9.15	1.33	5.76	1.01
U42718	7.09	1.77	7.03	2.25
U40730	8.50	1.93	5.24	2.07
U38764	11.83	1.88	4.12	1.08
U35159	4.22	1.42	5.39	1.33

## Appendix A Calculation of *K* for a non-sinusoidal field profile

The magnetic field profile in a hybrid planar undulator used in synchrotron light source and in free-electron laser facilities can in good approximation be considered a periodic magnetic field structure. On the contrary, the field shape may vary substantially from a perfect sinusoid. To precisely estimate *K*, it is mandatory to correctly take into account the actual field profile.

Starting from the well established equation of motion for relativistic electrons in a purely vertical magnetic field, where the dot (˙) indicates the derivative with respect to *z*,

it is natural to represent the period magnetic field with its Fourier series,

where only the  terms are present. Integrating equation (41) with respect to *z* gives

where  = . In the ultra-relativistic regime, it is possible to approximate the normalized component of the velocity, , with  and write the longitudinal component,  = , where β is the normalized speed of the electron. Simplifying this expression with the following approximation,

helps in explicitly writing its dependence as a function of the magnetic field,

Taking the average over a period, , gives the average component of the velocity along *z*,

Imposing the resonance condition for the radiation wavelength, λ, of an undulator,

where θ is the observation angle, it is then possible to recognize that

where finally

## Appendix B Fitting with logarithmic functions

While the relation between *K* and gap is well established as a clear exponential signature, its inverse function, gap *versus K*, is by far less popular and usually it is estimated numerically. The simple case of an exponential function,

has an analytical inverse function as it is easy to calculate,

On the contrary, the more general formulation

where  = , does not allow a simple inverse function formulation. Following the same generalization approach, a new logaritmic function can be defined for the inverse, where  = , as follows,

The symmetry between equations (52) and (53) is not perfect and this has important consequences. In equation (53) the multiplied term, , cannot be simplified in the argument of the  as is the case for the exponential function. The negative sign in front of equation (53) is equivalent to the negative sign in the argument of the exponential function in equation (50). In other words, it indicates that the function is not diverging but it is decaying while its argument increases. To fit the generalized logarithmic function it is convenient to manipulate equation (53) a bit further. After re-shuffling the multiplication term, the exponential function applies to both sides and simplifies the expression,

Applying the least-squares fit method to this new set of data gives the following equation,

where the parameter  is the only remaining non-linear term. A simple approach consists of evaluating  as a function of  and aiming for a minimum,

For this specific application it is easy to find the interval, , within which the best fit should be searched for.
